# Insights into Transfer of Supramolecular Doxorubicin/Congo Red Aggregates through Phospholipid Membranes

**DOI:** 10.3390/molecules29112567

**Published:** 2024-05-30

**Authors:** Anna Stachowicz-Kuśnierz, Paulina Rychlik, Jacek Korchowiec, Beata Korchowiec

**Affiliations:** Faculty of Chemistry, Jagiellonian University, Gronostajowa 2, 30-387 Krakow, Poland; paulina.rychlik@student.uj.edu.pl (P.R.); korchow@chemia.uj.edu.pl (J.K.)

**Keywords:** doxorubicin, Congo red, drug delivery, biomembranes, molecular dynamics simulations, Langmuir trough experiments

## Abstract

Doxorubicin (DOX) is a commonly used chemotherapeutic drug, from the anthracycline class, which is genotoxic to neoplastic cells via a DNA intercalation mechanism. It is effective and universal; however, it also causes numerous side effects. The most serious of them are cardiotoxicity and a decrease in the number of myeloid cells. For this reason, targeted DOX delivery systems are desirable, since they would allow lowering the drug dose and therefore limiting systemic side effects. Recently, synthetic dyes, in particular Congo red (CR), have been proposed as possible DOX carriers. CR is a planar molecule, built of a central biphenyl moiety and two substituted naphthalene rings, connected with diazo bonds. In water, it forms elongated ribbon-shaped supramolecular structures, which are able to selectively interact with immune complexes. In our previous studies, we have shown that CR aggregates can intercalate DOX molecules. In this way, they preclude DOX precipitation in water solutions and increase its uptake by MCF7 breast cancer cells. In the present work, we further explore the interactions between DOX, CR, and their aggregates (CR/DOX) with phospholipid membranes. In addition to neutral molecules, the protonated doxorubicin form, DXP, is also studied. Molecular dynamics simulations are employed to study the transfer of CR, DOX, DXP, and their aggregates through POPC bilayers. Interactions of CR, DOX, and CR/DOX with model monolayers are studied with Langmuir trough measurements. This study shows that CR may support the transfer of doxorubicin molecules into the bilayer. Both electrostatic and van der Waals interactions with lipids are important in this respect. The former promote the initial stages of the insertion process, the latter keep guest molecules inside the bilayer.

## 1. Introduction

**Scheme 1:**
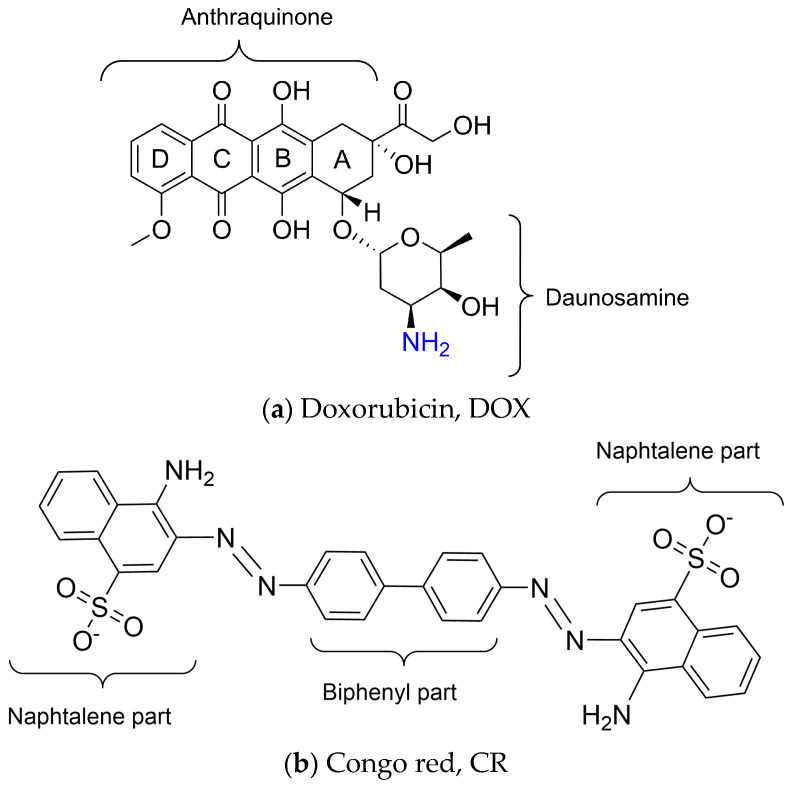
Molecules examined in the study and molecular fragments referred to in the discussion: doxorubicin (**a**) and Congo red (**b**). The protonable NH_2_ group in the daunosamine part of DOX is highlighted with blue color.

Doxorubicin (DOX, Scheme 1a, Figure S1a) is a popular anticancer drug from the anthracycline family [1]. Its main structural features include the daunosamine sugar moiety and the tetracyclic aglycone part, a derivative of anthraquinone, common to all anthracycline drugs [2]. The main cellular target of DOX is the cell nucleus, where it intercalates the anthraquinone part into DNA strands and blocks DNA/RNA synthesis and DNA repair, thus triggering programmed cell death [3,4,5]. The daunosamine group stabilizes minor groove binding and ensures water solubility. In addition to direct DNA binding, DOX causes damage to DNA and cell membranes by the generation of reactive oxygen species [6,7]. This universal mechanism of action allows doxorubicin to effectively treat various types of cancer. However, it is also responsible for the drug’s high toxicity, because DOX affects healthy cells as well [8,9]. Doxorubicin’s low toxicity index [10] prompted the search for targeted drug delivery systems, which would allow the lowering of the systemic DOX dose by selectively releasing the drug at its action site [11]. In the past few decades, many carrier systems have been proposed, e.g., liposomal formulations [12], functional carbon nanomaterials [13,14], covalent organics frameworks [15], boron nitrides [16], smart polymers [17] and polypeptides [18], or combined smart carriers [19,20].

In addition to the carrier systems listed above, synthetic dye molecules have been proposed as promising candidates for supramolecular drug carriers [21,22,23]. In recent years, Jagusiak et al. have reported on a possibility of employing aromatic azo dyes, specifically Congo red (CR, Scheme 1b, Figure S1c), as a carrier system for doxorubicin. CR is a planar molecule, built of a central biphenyl moiety and two substituted naphthalene rings, connected with diazo bonds. CR has been shown to self-assemble in water, forming elongated ribbon-shaped structures [24,25]. The driving force for the aggregation process is van der Waals interactions. The aggregates are further stabilized by HBs with water, allowing solvent molecules to screen electrostatic repulsion between the charged sulfonic groups. It was also shown that planar DOX molecules can be incorporated into CR aggregates by intercalating the anthraquinone part between CR molecules [25,26]. What is more, CR has the ability to selectively bind to immune complexes and proteins [27,28]. This feature can be used to load supramolecular DOX/CR complexes on drug-carrying proteins, e.g., albumin [29,30]. It has recently been shown that by using CR, it is possible to form a three-component carrier system containing albumin, CR, and DOX (which does not bind to albumin by itself) [31]. Since albumin accumulates in tumors [32], such a carrier may be used to selectively deliver DOX to the target site. Moreover, DOX was controllably released from the carrier in response to pH lowering, which is another promising feature, due to the known fact that the pH ranges from 5 to 5.5 inside neoplastic cells to 6.5 to 6.9 in the tumor’s microenvironment [17,20]. Controlled release of DOX was also reported in a different three-component drug–carrier system including DOX, CR, and a carbon nanotube [33]. CR was also used as a helper molecule, allowing the controllable release of DOX from biopolymeric carrier microspheres [34].

As mentioned above, the primary site of doxorubicin action is the cell nucleus. Therefore, one of the crucial aspects that needs to be addressed is the transfer through the cell membrane. It is known that DOX molecules enter the cell by passive diffusion [1]. In one of our previous studies, we have shown that the presence of CR in surrounding medium increases the ability of DOX to penetrate MCF7 breast cancer cells [35]. With the use of molecular dynamics (MD) methods, we have shown that, by incorporating neutral DOX molecules in supramolecular aggregates, CR precludes the precipitation of DOX, which by itself has a low solubility in water [36]. In this way, CR increases the bioavailability of neutral DOX and promotes transfer through bilayers. However, doxorubicin possesses a protonable NH_2_ group in the daunosamine ring, whose pKa value equals 8.6 [37]. Therefore, in the most therapeutically relevant, acidic pH, DOX will exist predominantly in the protonated state. For this reason, in this work, we revisit the question of membrane transfer in a mixed doxorubicin/Congo red system, taking into account the protonated form of doxorubicin (DXP, Figure S1b) and using a POPC bilayer as a universal membrane model. Although this membrane model is surely oversimplified, we are at present interested in elucidating general trends in doxorubicin/Congo red/lipids interactions before moving to more realistic membrane models. MD simulations are again used to probe the interactions in the studied system, as well as to examine the molecular-level mechanism and energetics of interactions between the membrane and DOX, DXP, CR, and their aggregates. The results are supplemented by Langmuir trough measurements for a model POPC monolayer.

## 2. Results

### 2.1. Langmuir Trough Experiments

**Figure 1:**
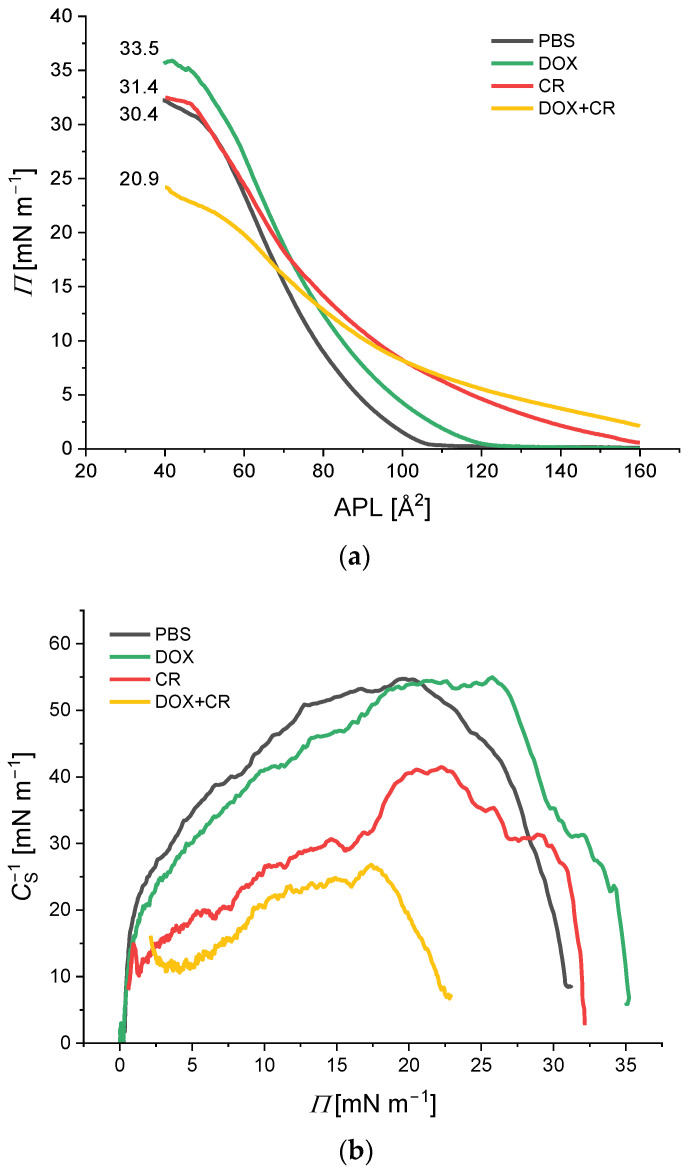
*Π*-*A* isotherms (**a**) and isothermal compressibility moduli, *C*_s_^−1^ (**b**) obtained at *T* = 310 K for POPC monolayer spread on pure PBS subphase (black), or doxorubicin (DOX, green), Congo red (CR, red), and DOX + CR (yellow) solutions. In panel (**a**), the value of collapse pressure, *Π*_coll_, is shown next to each curve in the small APL region.

Figure 1 shows *Π*-*A* isotherms (panel a) and plots of isothermal compressibility moduli, *C*_s_^−1^ (panel b), obtained at *T* = 310 K for POPC monolayers spread on a pure PBS subphase or on DOX, CR, or DOX + CR of equal volume. It can be seen that the presence of DOX in the subphase had little effect on the monolayer. The isotherm is uniformly shifted to larger areas per lipid (APL), but its shape and lipid compressibility remain unchanged. This behavior suggests that some association of DOX with the monolayer may occur, leading to increased APL, but it does not perturb the structure of the lipid film. When CR is present in solution, substantial changes are observed in the range of large APL values, corresponding to an expanded monolayer state. The isotherm is significantly shifted to larger areas and its slope is reduced, which is also reflected in a drop in *C*_s_^−1^ values. Such changes can be associated with monolayer fluidization, which can be an effect of the adsorption of CR molecules. Penetration of the monolayer by CR molecules would result in film expansion. An increase in APL as well as the presence of perturbing CR molecules would also hinder lipid packing, leading to monolayer fluidization. Interestingly, upon compression, the isotherm and *C*_s_^−1^ plot obtained on CR solution converge to curves obtained on pure PBS. This suggests that the adsorption of Congo red to the monolayer is reversible, and CR molecules are squeezed back to the subphase when the film is compressed. Finally, the isotherm obtained on DOX + CR solution exhibits similar changes in the large APL region, as the one measured on CR solution, showing that in this case, the interactions of CR and the lipid film are also present. However, the stability of the monolayer at increased *Π* is drastically reduced in this case: the surface pressure at the collapse point, *Π*_coll_, corresponding to the highest molecular packing in the monolayer, obtained on the DOX + CR solution is almost 10 mN m^−1^ lower than in the case of the unperturbed POPC monolayer. This suggests that in the presence of the DOX adsorption of Congo red, and possibly also doxorubicin to the monolayer, is not reversible. If these molecules remain in the monolayer at large *Π*, they may preclude chain alignment and lipid ordering and render withstanding high pressures impossible.

### 2.2. Molecular Dynamics Simulations

#### 2.2.1. Adsorption of DOX, DXP, CR, and Their Clusters to POPC Bilayer

Molecular dynamics simulations were employed to examine the interactions between the POPC bilayer and DOX, DXP, and CR. For this purpose, either individual molecules or their aggregates of 2–6 molecules were put in the vicinity of pre-equilibrated POPC bilayers with 100 and 225 lipids per leaflet (Figure S2). For each system, three independent models were constructed and simulated at *T* = 310 K and *p* = 1 atm for 280–520 ns. The outcomes of the simulations are summarized in Table 1. In what follows, one replica was chosen for each system for detailed discussion. The final states of these systems are shown in Figure S3. 

In summary, neutral doxorubicin in the form of single molecules (DOX1) spontaneously and reversibly crossed the bilayer’s headgroup region. In simulations with clusters of two (DOX2) or three (DOX3) molecules, the clusters came apart in water and DOX adsorbed to the bilayer as individual molecules. Adsorption of aggregated DOX was not observed in any simulation, even when the harmonic wall potential was applied to keep the DOX clusters whole. On the contrary, protonated doxorubicin remained in the aggregated form in water. Single molecules (DXP1) as well as two-molecule clusters (DXP2) entered beneath the hydrophilic bilayer region. The latter preserved the aggregated form inside the bilayer. Congo red molecules also remained clustered for the whole simulation time and passed the headgroup region in the aggregated form. Single molecules (CR1) and clusters of two molecules (CR2) readily adsorbed to the smaller bilayer model (100 lipids per leaflet). Adsorption of a three-molecule cluster (CR3) was only observed for the larger bilayer (225 lipids per leaflet). Once in the membrane, DXP and CR clusters remained inside until the end of each simulation. Finally, in the mixed DOX + CR systems, adsorption was only observed in one simulation of the 1:1 DOX:CR cluster (DOX1CR1). About 300 ns after adsorption, the DOX1CR1 cluster dissolved, and the two molecules separated from each other. Two cases of headgroup penetration were observed in the mixed DXP + CR systems. Unlike DOX1CR1, the adsorbed DXP1CR1 clusters did not dissociate inside the bilayer.

**Figure 2:**
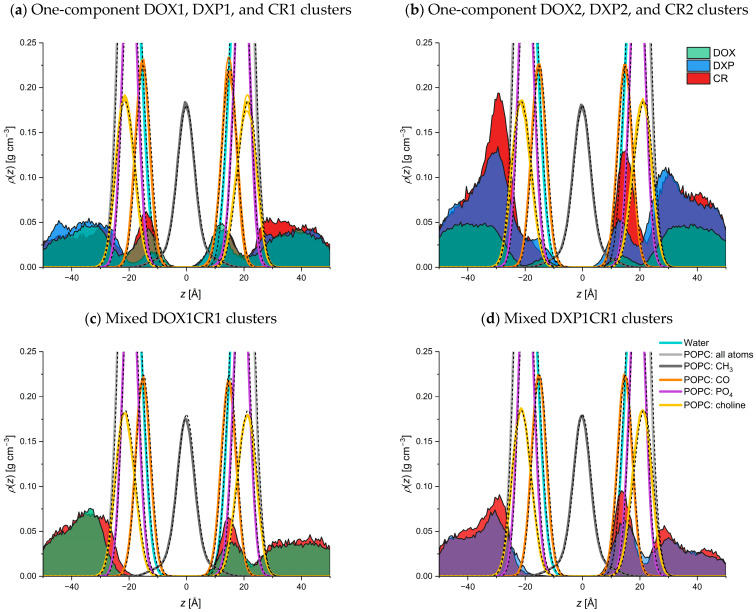
Density profiles along bilayer normal, *ρ*(*z*), for selected system components, obtained after averaging 280–470 ns of adsorption simulations with 100 lipids/leaflet POPC bilayers. Results for single guest molecule systems (DOX1, DXP1, and CR1, (**panel a**)), clusters of two identical molecules (DOX2, DXP2, CR2, (**panel b**)), and mixed two-molecule clusters (DOX1CR1, (**panel c**), and DXP1CR1, (**panel d**)) are compared. All curves are averages over three independent simulations (310 K, 1 atm). Colored solid lines represent water (cyan), all bilayer atoms (light grey), and selected bilayer components: *sn*-1 and *sn*-2 chain terminal CH_3_ groups (dark grey), *sn*-1 and *sn*-2 carbonyl groups (orange), phosphate groups (magenta), and choline (yellow). Dashed black lines mark averaged profiles obtained for clean POPC systems. Shaded areas represent the profiles for guest molecules: neutral doxorubicin (green), protonated doxorubicin (blue), and Congo red (red). The profiles for guest molecules were magnified ten times.

In order to illustrate the above description, variations in the *z* coordinate of the center of mass, *R*_COM_(*z*), of bilayers and guest molecules were plotted as a function of the simulation time. The results are presented in Figures S4–S6 of the Supplementary Materials. The plots show that in the aqueous phase, guest species associate with the bilayer surface for tens to about a hundred nanoseconds. This behavior seems most pronounced for medium-sized (2–3 molecules) clusters, especially those containing the charged CR or DXP species. This behavior can be verified by plotting the partial density profiles of selected system components along the bilayer normal, *ρ*(*z*). They were extracted from full adsorption simulations, i.e., the initial 50 ns of each simulation was discarded (equilibration), and the plot was averaged over the remaining simulation time (230–470 ns). Plots for all simulations with a 100 lipid/leaflet bilayer are gathered in Figures S7 and S8. Magnified profiles for one- and two-molecule clusters are compared in Figure 2. Distinct maxima in the proximity of the bilayer show that the charged DXP and CR molecules (blue and red shaded areas) associate with the bilayer surface to a larger extent than neutral DOX molecules (green areas). Interestingly, in systems with mixed DXP + CR clusters, the tendency to associate with the bilayer surface is clearly higher than in DOX + CR systems. Increased bilayer association of two- and three-molecule clusters with respect to systems containing single guest molecules, as well as in DOX2 and DOX3 (disintegrated clusters), can also be seen. 

**Figure 3:**
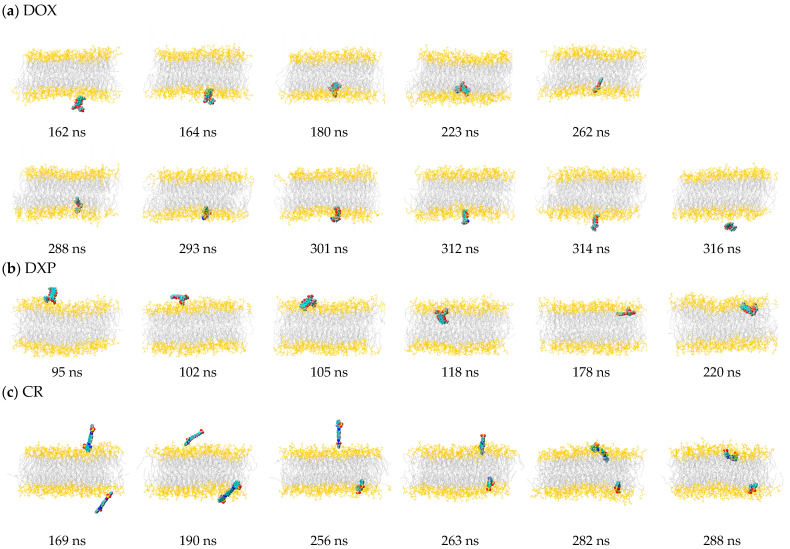
Snapshot sequences illustrating entry of single DOX (**a**), DXP (**b**), and CR (**c**) molecules into POPC bilayer (100 lipids/leaflet, 310 K). Snapshots are labeled with a timestamp corresponding to the time elapsed from the beginning of each simulation. Lipid headgroups are drawn with yellow lines, lipid chains are depicted in grey. DOX, DXP, and CR are depicted with van der Waals spheres, colored according to element: carbon—cyan, oxygen—red, nitrogen—blue, sulfur—yellow, hydrogen—grey. Water and counterions are omitted for clarity. In DOX and DXP systems, the second guest molecule is also omitted.

The *R*_COM_(z) plots in Figures S4–S6 also show that bilayer entry is fast for single DOX, DXP, and CR molecules. In these cases, less than c.a. 30 ns passes from the moment when the COM of the guest species first crosses the bilayer boundary, until it reaches its final position inside the bilayer. The transfer of aggregated clusters through the headgroups is slower and takes 50–150 ns. The molecular details of bilayer entry by single DOX, DXP, and CR molecules are illustrated in the form of snapshot sequences in Figure 3.

For both doxorubicin forms, the whole insertions process took c.a. 30 ns. In all instances of bilayer penetration by DOX (11 cases), this molecule first inserts the anthraquinone ring between lipid headgroups, as exemplified in Figure 3a. The ring is transferred to the other side of the headgroups in vertical orientation, i.e., parallel to the bilayer normal. It is followed by the rest of the molecule. After reaching the membrane interior, the molecule rotates, and orients along the bilayer plane. When in the membrane, DOX molecules occasionally insert the anthraquinone part in between lipid tails. As stated above, DOX adsorption is reversible. The DOX release mechanism is the reverse of the entry process. In orientations with an anthraquinone ring in between lipid tails, the daunosamine part of the molecule is directed towards the headgroups. Such an orientation may lead to the vertical transfer of the molecule outside the bilayer. On the other hand, DXP first interacts with the bilayer surface through the protonated NH_3_^+^ group in the daunosamine part. Inspection of the trajectories reveals that DXP molecules often associate with the bilayer surface by inserting the daunosamine part between the headgroups, with the rest of the molecule oriented in the bilayer plane. An example of such a structure is given in Figure 3b (snapshot at 102 ns). Insertion into the bilayer proceeds through molecule rotation. Protonated daunosamine remains anchored in the headgroups, while the anthraquinone ring rotates towards the chains. Inside the membrane, the DXP molecule often adopts orientations mirroring the one from the aqueous side, with the daunosamine part inserted between the headgroups, and the rest of the molecule oriented in the bilayer plane from the hydrophobic side (snapshot at 178 ns, Figure 3b). Similarly to neutral DOX, in the case of DXP, insertion of the anthraquinone ring between the tails was observed. However, in the case of DXP, the release of DXP was not recorded. Congo red molecules approach the bilayer in vertical orientation (Figure 3c). The entry process begins with the insertion of one of the naphthalene rings between the headgroups. Once this ring is transferred to the other side of the headgroups and begins to interact with the chains, the CR molecule remains associated with the bilayer. The rest of the molecule slowly rotates, and the final transfer beneath the headgroups occurs with the molecule oriented in the bilayer plane.

Adsorption of DXP2, CR2, and CR3 clusters is shown in Figure S9. For two-molecule DXP2 and CR2 clusters, the mechanism remains similar to the single-molecule cases. However, in the case of CR3, a different mechanism was observed. The cluster approached the bilayer in the horizontal orientation (parallel to the bilayer). Next, the molecule that was closest to the bilayer started penetrating the headgroup region. At the same time, it partly separated from the cluster. This molecule then acted as an anchor, keeping the cluster associated with the bilayer surface and allowing the slow transfer of the whole cluster beneath the headgroups. The whole process was considerably slower than in other systems and took c.a. 150 ns (Figures S5 and S9). 

**Figure 4:**
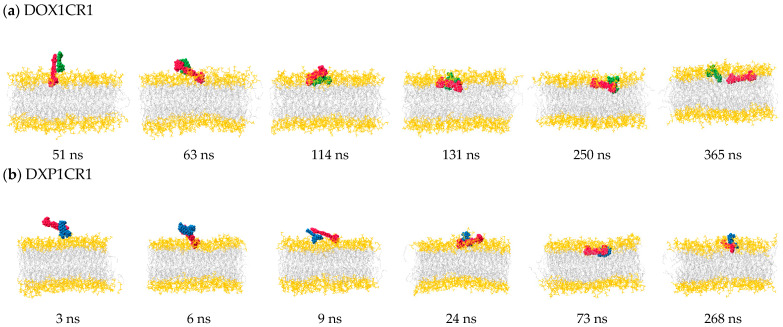
Snapshot sequences illustrating adsorption of mixed DOX1CR1 (**a**) and DXP1CR1 (**b**) clusters onto POPC bilayer (100 lipids/leaflet, 310 K). Snapshots are labeled with a timestamp, corresponding to the time elapsed from the beginning of each simulation. Lipid headgroups are drawn with yellow lines, lipid chains are depicted in grey. DOX, DXP, and CR are depicted with green, blue, and red spheres, respectively. Water, counterions, and the second cluster in each system are omitted for clarity.

Finally, the entry of mixed DOX1CR1 and DXP1CR1 clusters is illustrated in Figure 4. In the first step, the clusters associate with the bilayer surface. A characteristic feature of this stage is a structure in which one of the naphthalene rings of CR is inserted between the headgroups and doxorubicin (neutral or protonated) remains outside. Examples of such structures are shown in Figure 4a (snapshot at 51 ns) and Figure 4b (snapshot at 6 ns).

This type of structure has been observed many times in all simulations of mixed clusters. Although the clusters may remain in contact with the bilayer surface for tens of nanoseconds, association is reversible. Despite numerous instances of naphthalene insertion, there were only three cases of whole cluster transfer beneath the headgroups—one in the DOX1CR1 systems and two in the DXP1CR1 systems. In all cases, the cluster first rotated to obtain a horizontal orientation on the bilayer surface, and next was slowly transferred to the heads/tails boundary. An important difference in DOX + CR and DXP + CR systems emerged when the clusters were already inside the membranes, namely, after c.a. 300 ns, the DOX1CR1 cluster dissociated into individual molecules. On the other hand, the DXP + CR cluster remained in the aggregated form. 

**Figure 5:**
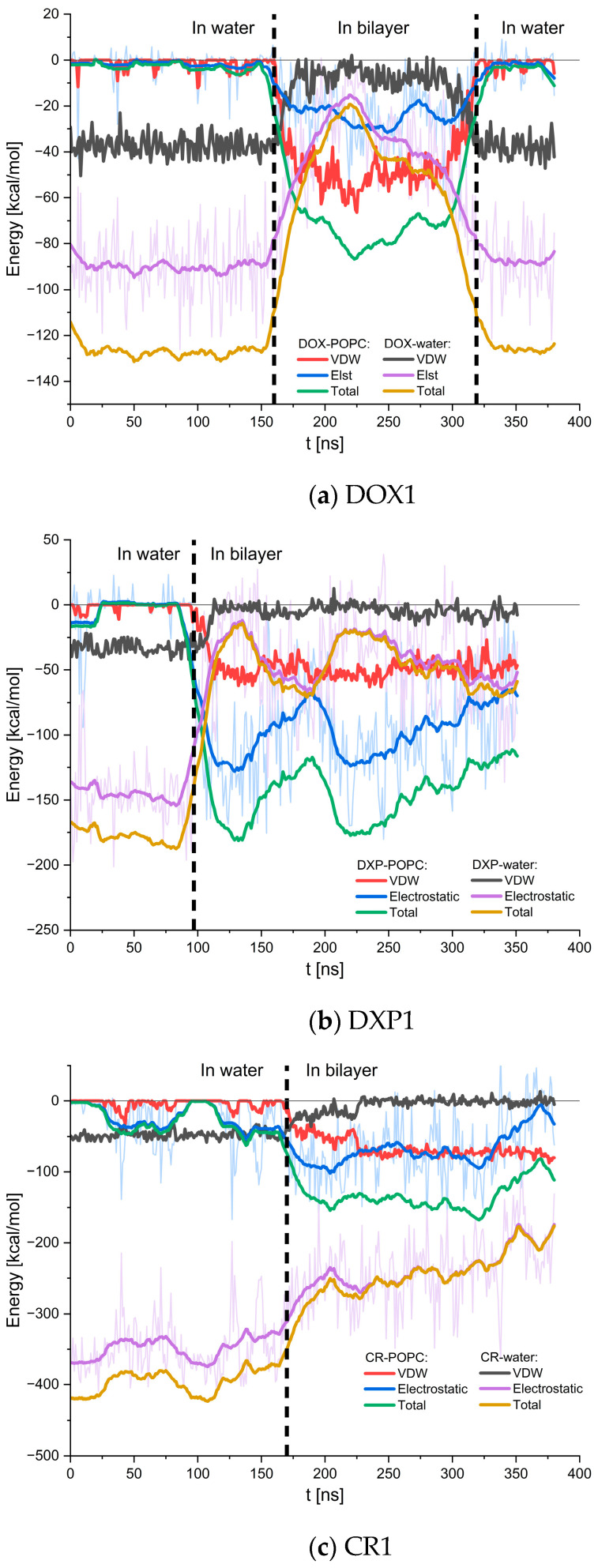
Variations in interaction energies between single DOX (**a**), DXP (**b**), and CR (**c**) molecules and POPC bilayer or water, accompanying entry of guest molecules into the bilayer. Van der Waals (red, dark grey) and electrostatic (blue, violet) contributions, and their sum (green, yellow) are plotted. For electrostatic and total energies, smoothed curves are plotted, due to large fluctuations. Unsmoothed energies are shown as light lines in the background. Vertical dashed lines mark the time when the COM of the guest molecule crosses the membrane boundary.

In order to reveal the driving forces for cluster entry to the bilayers, the variations in interaction energies accompanying this process can be analyzed. Figure 5 presents the plots of nonbonding contributions to the potential energy function (CHARMM force field) between single guest molecules and either POPC or water calculated along adsorption simulations. It can be seen that in the aqueous phase, when the molecules are away from the bilayer surface, the energy of their interaction with the membrane falls to zero, and the energy of the interaction with water attains a steady average value. As expected, the magnitude of guest molecule–water interactions increases with the increasing net charge of the solute. In a simulation of the CR1 system, between c.a. 20 and 80 ns, the guest molecule associates temporarily with the bilayer surface. In the left part of Figure 5c, it can be seen that electrostatic attraction between the charged CR molecules and lipid headgroups is responsible for this behavior. It can also be seen that association with the bilayer surface leads to dehydration of the guest molecule, which is reflected in the rise in the CR–water interaction energy. A similar pattern can be seen in the beginning steps of the DXP1 system simulation.

In all panels of Figure 5, vertical dashed lines correspond to the moments when the COM of the guest molecule first crosses the bilayer boundary. Their positions were obtained from *R*_COM_(z) plots (Figures S4–S6). As guest molecules cross this region, abrupt changes in the interaction energies occur. The dominant factor driving entry to the bilayer varies between the studied molecules. For all guest molecules, van der Waals (VDW) attraction between the guest and POPC is an essential factor. The contribution of electrostatic energy depends on the guest molecule’s net charge. For the neutral DOX molecule, the VDW energy is the main contribution to the DOX–membrane interaction. The maximum VDW stabilization in DOX–POPC interactions corresponds to the complete transfer of the DOX molecule beyond the headgroup region (Figure 3a, snapshot at 223 ns). Penetration of the hydrophobic bilayer core is, however, accompanied by the dehydration and loss of stabilizing DOX–water interactions. Therefore, the molecule attains a vertical orientation with an anthraquinone ring inserted between the chains and daunosamine part in contact with headgroups and water. In the discussed system, such an orientation led to DOX release. However, in other replicas, DOX periodically changed orientation from completely embedded to vertical and remained in the membrane. In the case of the charged DXP molecule, electrostatic interactions with POPC are stronger than VDW. Entry of DXP begins with a drop in electrostatic DXP-POPC energy. It corresponds to the association of the DXP molecule with the surface of the bilayer (Figure 3b, snapshot at 102 ns). However, as soon as the anthraquinone ring comes into VDW contact with the lipids, the molecule is rotated and the ring is transferred to the hydrophobic core (Figure 3b, snapshot at 118 ns). This orientation corresponds to the first minimum on the DXP-POPC energy plot. Similarly to the DOX system, stabilizing interactions with water enforce the periodic return of the anthraquinone ring to the heads/tails boundary region (Figure 3b, snapshot at 178 ns). In the CR1 system, bilayer entry is preceded by pre-association with the membrane surface, stabilized by the electrostatic interactions of CR with lipids (Figure 3c, snapshot at 169 ns). Electrostatic interactions also promote the initial stage of CR entry, which corresponds to transfer through the outermost headgroup region. At the same time, the aromatic rings of the guest molecule gradually come into contact with the lipid tails and VDW interactions start to play a role (Figure 3c, snapshot at 190 ns). CR contains three large aromatic groups; therefore, the energy of its VDW interaction with the lipids is roughly two times bigger than for DOX-POPC (both molecules have the same number of atoms). In this system, the electrostatic and VDW interactions with lipids are of similar strength. On the other hand, for this molecule with a net charge of −2, interactions with water are also strong. Therefore, CR does not penetrate the hydrophobic core of the bilayer. Instead, it adopts a flat orientation on the heads/tails boundary. A drop in the electrostatic energies of both CR-POPC and CR–water interactions in the final stages of the simulation corresponds to binding a sodium ion by CR (highlighted in black in Figure S3).

**Figure 6:**
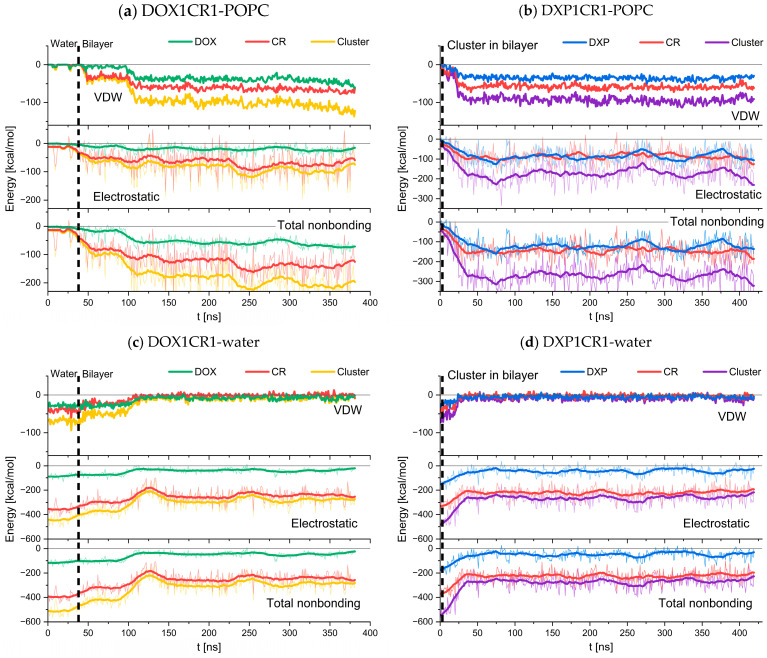
Variations in interaction energies between DOX1CR1 (**a**,**b**) and DXP1CR1 (**c**,**d**) clusters and POPC bilayer (**a**,**c**) or water (**b**,**d**), accompanying the clusters’ entry into the bilayer. Van der Waals (top) and electrostatic (middle) contributions, and their sum (bottom) are plotted separately. Each energy term is further split into individual DOX (green), DXP (blue), and CR (red) contributions, and their sum (yellow, violet). For electrostatic and total energies, smoothed curves are plotted, due to large fluctuations. Unsmoothed energies are shown as lighter lines in the background. Vertical dashed lines mark the time when the COM of the cluster crosses the membrane boundary.

Energies obtained for the mixed DOX + CR and DXP + CR clusters are presented in Figure 6. In these systems, adsorption occurred at the very early stage of the simulations; therefore, the plots for the second cluster in each system (which remained in water) are given in Figure S10. It can be seen there that in the DOX1CR1 system (Figure S10a), electrostatic attraction between CR and the lipid headgroups is the main factor responsible for the pre-association of the cluster with the bilayer surface. CR-POPC interactions are also the driving force for the early stages of the entry process, corresponding to structures with one of the naphthalene rings of CR inserted between the headgroups (Figure 4a, snapshot at 51 ns). DOX-POPC interactions become significant only after the cluster is rotated to the bilayer plane (Figure 4a, snapshot at 114 ns), and promote its transfer beneath the headgroups. After that, the interaction energies stabilize until c.a. 250 ns, when DOX and CR molecules start separating (Figure 4a, 365 ns). Cluster dissociation can also be seen as the vanishing of intra-cluster interactions, manifested in the plot of the DOX-CR interaction energy, as shown in Figure S11. On the other hand, in the DXP + CR system, the DXP molecule is responsible for reversible cluster adsorption to the membrane surface. The minima in the plots of the DXP–lipid energies, shown in Figure S10, correspond to structures with the DXP molecule lying flat on the bilayer surface, with the protonated daunosamine embedded in the headgroup region, and the CR molecule located flat on top of DXP. In contrast, both molecules are involved in stabilizing interactions with lipids, when bilayer entry takes place. Both electrostatic and VDW energies contribute to a comparable degree to promote cluster insertion beneath the headgroups. In the membrane, DXP and CR molecules remain aggregated. The DXP-CR interaction energy shown in Figure S11 remains relatively stable until the end of the simulation. A temporary rise in the cluster–lipid energies at c.a. 270 ns corresponds to the brief exposure of DXP to water, as shown in Figure 4b.

To sum up this part of the study, it can be stated that all of the examined molecules and their dimers adsorb to the POPC bilayers, although the molecular details of this process are different in each system. Not surprisingly, the net charges of the guest molecules are the main factor determining the mechanism of bilayer entry. Molecules possessing a charged functional group (NH_3_^+^ in DXP and SO_3_^−^ in CR) are attracted to the bilayer surface. Due to strong electrostatic stabilization, they tend to embed the charged group in the headgroup bilayer region. However, for crossing the headgroup layer, VDW interaction between the guests and lipids is essential. All guest molecules contain large aromatic parts, which are stabilized in the hydrophobic bilayer core. VDW interactions have smaller fluctuations and faster decay with distance than electrostatic ones; therefore, guest molecules need to remain in contact with the lipid tails in order to maintain them. By residing near the heads/tails boundary, they also preserve most of the stabilizing electrostatic interactions with the headgroups and part of the interactions with water. In light of the above observations, it can be expected that CR, possessing both a charged group and large aromatic portion in its’= structure, will exhibit a considerable affinity for the membrane. This affinity could be a root cause of the shift in the isotherm to larger areas, observed for the POPC monolayer in Langmuir trough experiments in the presence of CR. However, it has to be stressed that bilayer entry is accompanied by solute dehydration and the loss of stabilizing interactions with water. This energy cost is especially big for the charged DXP and CR molecules. It is probably responsible for the relatively low frequency of adsorption events, which was only up to 50% of the guest molecules (Table 1).

#### 2.2.2. Behavior of Guest Species Inside POPC Bilayer

The goal of the second part of this study was to examine the fate of guest species inside the POPC bilayer, as well as their influence on the membrane structure. Since few instances of bilayer adsorption were recorded for the aggregated clusters, additional systems were prepared by manually pulling the clusters into the bilayer COM. Single molecules and selected clusters were chosen for analysis (see *Computational Details*). A bilayer model with 100 lipids/leaflet was used. After equilibration, all artificial forces were removed, and the systems were simulated for 100–400 ns at *T* = 310 K and *p* = 1 atm. The results presented below were obtained by averaging over all systems with a cluster of a given composition inside the bilayer. This includes parts of adsorption simulations successive to bilayer entry, as well as the additional systems obtained by pulling. In this way, 3–6 independent simulations were obtained for each of the discussed cluster compositions.

The outcomes of the additional simulations were in agreement with the adsorption calculations, namely, the DOX2 and DOX3 clusters disintegrated over time. In two systems, one DOX molecule left the bilayer. Among the DOX1CR1 clusters, one disintegrated and the remaining two stayed aggregated. On the other hand, in the DOX2CR1 systems, two disintegrated and one stayed aggregated. For clusters containing DXP, the tendency to disintegrate was much smaller. Only one case of DXP3 cluster dissociation was observed. In all the remaining systems, the clusters stayed in the aggregated form.

**Figure 7:**
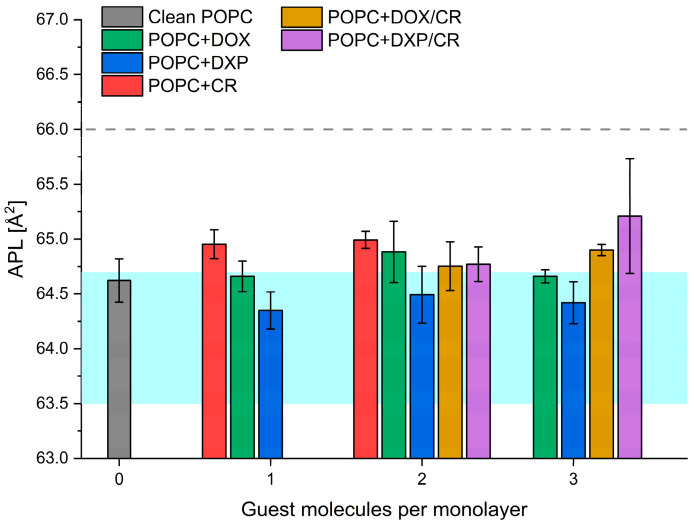
Averaged areas per lipid (APL) obtained from simulations of clean POPC bilayer (grey), and bilayers with CR (red), DOX (green), DXP (blue), DOX + CR (yellow), or DXP + CR (purple) clusters inside. Experimental APL estimate [38] is shown with the horizontal dashed line. The range of the literature APL values obtained from MD simulations [39] is marked with a cyan area. Each entry is the average of 3–6 individual simulations. Error bars were estimated as standard deviation between individual simulations.

One of the important characteristics of a lipid bilayer is the area per lipid. For a pure POPC bilayer at 310 K, the experimental APL estimate equals 66 Å^2^ [38]. As far as the MD simulations are concerned, values obtained at 310 K with the use of CHARMM force fields fall in the range of 63.7–64.7 Å^2^ [39]. The MD values are therefore underestimated. However, the comparison is not straightforward, since the APL cannot be measured directly, and the MD values are scattered. Variations in the APL accompanying the guest molecules’ adsorption are included in *R*_COM_(*z*) plots, presented in Figures S4–S6. Along the simulations, the APL values fluctuate in the range roughly corresponding to the literature MD values. No significant APL changes can be observed upon guest species entry (indicated by vertical dashed lines). This is not surprising, since the clusters studied in this work are small in comparison to the bilayer size and the change in the APL induced by cluster insertion is obscured by natural APL fluctuations. Some trends can be observed when the averaged APL values are analyzed (Figure 7). In the case of the one-component clusters, when the bilayers with CR, DOX, and DXP are compared, the APL decreases in this series for all cluster sizes. For CR clusters, the areas are increased with respect to the clean POPC bilayer. On the contrary, when the DXP clusters are inserted, the areas decrease. In systems with DOX, intermediate behavior was observed. Interestingly, changes in the APL do not increase much with the increasing cluster size. In systems with mixed clusters, the APL is always increased with respect to the clean bilayer. For the DOX1CR1, DOX2CR1, and DXP1CR1 systems, the magnitude of this increase is smaller than that caused by a single CR molecule. In the DXP2CR1 system, the increase is also dependent on cluster geometry, namely, it is smaller when the DXP molecules are located on opposite sides of the CR molecule, and larger if they are located on the same side (Figure S12). These differences are the source of the large error bars reported in this case.

**Figure 8:**
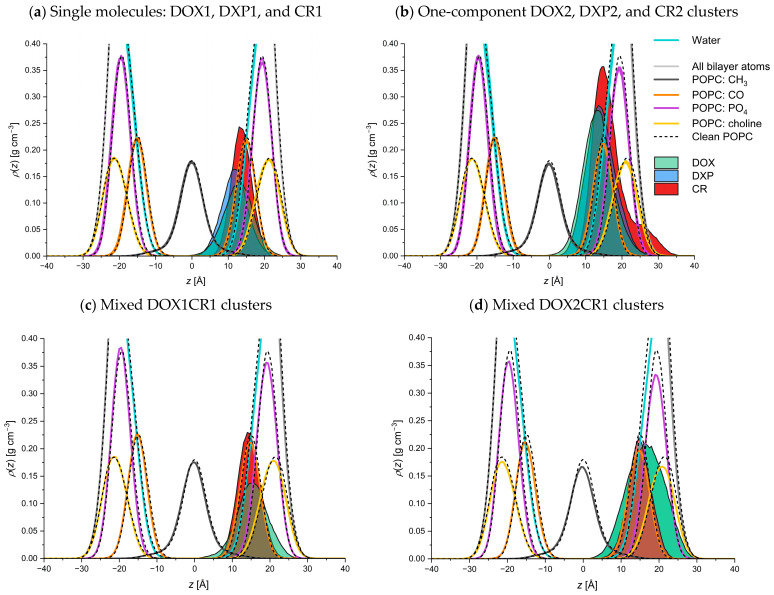

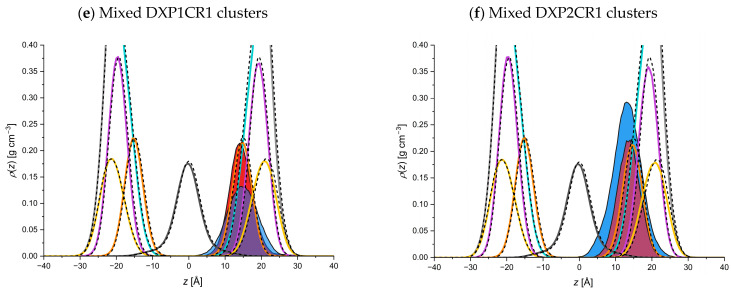
Density profiles along bilayer normal for selected system components, extracted from simulations with clusters inside the bilayers. Results for single guest molecules (**panel a**), clusters of two identical molecules (**panel b**), and mixed clusters of two (**panels c**,**d**) or three (**panels e**,**f**) molecules are compared. All curves are averages over 3–6 independent simulations (310 K, 1 atm). Colored solid lines represent water (cyan), all bilayer atoms (light grey), lipid chain terminals CH_3_ groups (dark grey), lipid carbonyl groups (orange), phosphate groups (magenta), and choline (yellow). Dashed lines mark profiles obtained for clean POPC. Shaded areas represent the profiles for guest molecules: neutral doxorubicin (green), protonated doxorubicin (blue), and Congo red (red). The profiles for guest molecules were magnified ten times.

Density profiles along the bilayer normal allow tracking the location of guest species inside the bilayers. In panels a and b of Figure 8, *ρ*(*z*) plots obtained for single molecules and one-component dimers are presented. It can be seen that the profiles for CR are sharper than for DOX and DXP. This indicates that the location of CR is more conserved throughout the simulation. The conserved structure corresponds to CR molecules oriented in the bilayer plane on the heads/tails boundary. It preserves the stabilizing electrostatic interactions between the polar substituents in CR with headgroups and water. CR molecules do not penetrate the hydrophobic bilayer region, as shown by the profiles decaying together with those of carbonyl and phosphate groups. On the other hand, DOX and DXP molecules enter deeper into the bilayer. Throughout the simulation, they reversibly insert the anthraquinone ring into the chain region. This behavior is reflected by *ρ*(*z*) curves extended towards the bilayer core, reaching as far as the bilayer center. The location of the CR molecules is not changed in systems with mixed clusters (Figure 8, panels c–e). On the other hand, the location of DOX and DXP is shifted towards the bilayer surface. This means that interactions between CR and DOX/DXP retain the drug molecule close to the heads/tails boundary. However, in the case of DOX + CR systems, this behavior can be regarded as temporary due to cluster disintegration. After breakdown, the pattern observed for individual DOX and CR molecules is restored (e.g., broadened profiles for DOX in DOX2CR1 system). On the contrary, in DXP + CR systems, where the clusters remain in the aggregated form, the profiles for DXP remain sharp. Interestingly, in the DXP2CR1 system, the penetration of the hydrophobic core is also increased. This suggests that bilayer perturbation caused by the insertion of this otherwise bulky cluster is reduced by moving DXP towards the tails.

**Figure 9:**
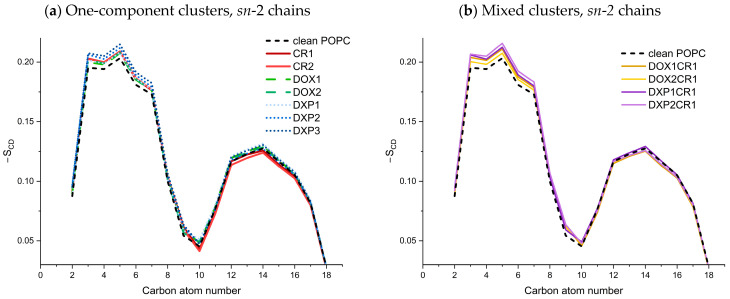
Order parameter for *sn*-2 lipid chains in POPC bilayers with one component (**a**) and mixed (**b**) DOX/DXP/CR clusters. The values were obtained by averaging over all systems with a given cluster inside the bilayer (3–6 systems). Results for clean POPC bilayer are shown with dashed lines.

More insight into the influence of guest clusters on lipid packing can be gained by the analysis of the chain order parameters, *S*_CD_. The results obtained for *sn*-2 chains are presented in Figure 9. The *S*_CD_ values obtained for *sn*-1 chains are given in Figure S13. It can be seen that the presence of guest species has a moderately ordering effect on the lipids. However, this effect is only pronounced near the heads/tails boundary. The *S*_CD_ values are increased for carbon atoms in the first part of the chain (before the C9–C10 double bond). For the terminal parts, they either converge to the ones obtained for pure POPC bilayers or, in the case of CR, they are lowered. The ordering effect is most pronounced for systems that contain DXP molecules. This suggests that interactions with the positively charged DXP molecule are important in this case. It can be hypothesized that DXP molecules screen electrostatic repulsion between lipid phosphate groups and in this way allow for increased lipid packing on the heads/tails boundary. This would explain the APL reduction observed for systems with DXP.

## 3. Discussion

In the present study, mutual interactions between POPC membranes and doxorubicin/Congo red are analyzed by means of molecular dynamics simulations and Langmuir trough measurements. A possible role of CR as a carrier, promoting the transfer of doxorubicin through the membrane, is examined. 

Both the Langmuir measurements and simulations show that all of the examined species interact with POPC membranes, but each in a different fashion. CR has a strong affinity towards POPC membranes. In simulations, spontaneous entry beneath the headgroups region by individual CR molecules and their cluster was observed. *Π*-*A* isotherms, showing a substantial shift towards larger APL in the presence of CR, confirm that membrane adsorption occurs. However, the isotherm shift is most pronounced at large APL values. This observation points towards a conclusion that the membrane has to be in a relaxed state in order for CR entry to occur. This is in agreement with our previous simulations with ordered DPPC membranes [35], where spontaneous entry was not observed. MD simulations show that the affinity of CR towards the membrane is rooted in the cooperation of electrostatic and van der Waals forces. Electrostatic attraction between charged CR molecules and lipid headgroups is responsible for the association with the bilayer surface and initial stages of crossing the headgroups region. On the other hand, van der Waals interactions preclude the release of adsorbed CR molecules back to the aqueous phase. CR did not penetrate the hydrophobic bilayer core. Regardless of whether it adsorbed from water or was pulled inside the bilayer core, its final location was on the heads/tails boundary, which is a well-known membrane binding site for amphiphilic molecules [40]. Once inside, CR has a fluidizing effect on the membranes, as shown by the drop in monolayer compressibility moduli and APL increase in simulations. 

Passive diffusion has been identified as the main route of doxorubicin’s entry into the cells [41]. Therefore, the transfer of this drug through lipid membranes is a well-studied topic [37,42,43,44]. Doxorubicin–membrane interactions were also proposed as an important factor contributing to drug resistance mechanisms [45,46,47]. Doxorubicin has a strong affinity towards cell membranes [48]. This affinity is especially pronounced for membranes containing anionic lipids, as can be expected for the protonated form of the drug. In addition to ionic attraction, interactions of the drug with the hydrophobic core of model membranes have been documented [49] as well as a condensing effect of doxorubicin on zwitterionic bilayers [50]. The results obtained in this work are in general agreement with the picture emerging from the above experimental studies, as well as the MD results reported in the literature for zwitterionic lipids [50,51,52,53,54,55]. Neutral DOX in the form of single molecules spontaneously and reversibly penetrated the POPC bilayer. Comparison between the neutral and protonated forms shows that DXP has a higher affinity to POPC. DXP also exhibits a smaller tendency to leave the bilayer. The increased affinity of DXP towards POPC can be explained by two factors. The first is a simple electrostatic interaction between the charged DXP molecules and zwitterionic lipid heads. In this respect, the observed pre-association with the bilayer surface, with the insertion of the daunosamine part between the headgroups, seems to be an important intermediate state. The electrostatic attraction of DXP and phosphate groups in POPC may also be responsible for the reduced tendency to leave the membrane. The second factor may be connected with the condensing effect of DXP on the lipids near the heads/tails boundary. DXP may lead to APL reduction by screening the electrostatic repulsion between PO_4_ groups, which in turn may hinder the release of the drug. Both DOX and DXP molecules insert their anthraquinone part between the tails, which is consistent with the proposed mechanism of doxorubicin transfer by flip-flop [56].

As far as mixed drug–carrier systems are concerned, the results of this study suggest that CR may assist bilayer penetration by DOX and DXP. Similarly to CR-only systems, electrostatic interactions between CR and lipid headgroups are responsible for attracting mixed clusters to the bilayer surface and assisting the initial stages of cluster adsorption. In this respect, the active role of CR in promoting the transfer of doxorubicin through the headgroups region is an important finding. In our previous study, where DPPC membranes were used, we concluded that the main role of CR in promoting DOX uptake by MCF7 cells lies in the change in the aggregation patterns of DOX in water [35], namely, in the presence of CR, the bioavailability of DOX was increased by incorporating DOX molecules into the DOX + CR clusters and increasing the drug’s solubility. Spontaneous penetration of the DPPC membrane by either CR or DOX + CR clusters was not observed in that study. The present study shows that with more fluid membranes, CR may assist the adsorption of doxorubicin molecules to the bilayer. This effect may be especially important for the neutral form of the drug, for which electrostatic interactions with the membrane are otherwise small.

However, promoting bilayer adsorption does not imply an immediate increase in doxorubicin transfer across the membrane. On the contrary, simulations of mixed clusters inside the POPC bilayer show that interactions between CR and doxorubicin constrain the drug molecule close to the heads/tails boundary. This effect seems especially important for the protonated form of the drug. It may also be responsible for the observed reduction in monolayer stability observed at high surface pressures in the Langmuir trough experiments. On the other hand, for neutral DOX molecules, cluster decomposition observed at longer time scales may revert this effect and restore the behavior observed for single DOX molecules, i.e., insertion of the anthraquinone to the tails region. 

The present study underlines the three most important features of the interactions between doxorubicin/Congo red drug–carrier system and POPC membranes: (i) electrostatic interactions between the drug, carrier, and lipids, (ii) van der Waals interactions between the aromatic parts of the drug–carrier complex and lipids, and (iii) membrane fluidity. Electrostatic interactions, which drive the initial penetration steps, are determined by the protonation state of doxorubicin. The MD simulations performed here show that the protonated form, dominant at neutral and acidic pH, has an increased potential to adsorb to the bilayer. However, penetration of the headgroups region is not sufficient to cross the membrane barrier. The next step, involving the flip-flop of the drug molecule, is strongly favored for the neutral form. The observed increase in the penetration of MCF7 cells by doxorubicin in the presence of CR may therefore be related to the ability of CR to increase the adsorption of the neutral DOX form and subsequent release of the drug molecules. Alternatively, the protonation equilibrium of doxorubicin could be considered. Due to the inhomogeneous character of bilayer systems, the effective pH inside the bilayer may differ from the pH of the aqueous phase. It cannot be ruled out that doxorubicin undergoes a deprotonation reaction inside the membrane, which would allow its transfer across the bilayer. Further research on this topic is needed to test this hypothesis. The possible role of Congo red as a proton acceptor also needs to be investigated. 

## 4. Materials and Methods

### 4.1. Materials

Synthetic 1-palmitoyl-2-oleoyl-*sn*-glycero-3-phosphocholine (POPC, 99% pure), Congo red (CR, 96% pure), phosphate-buffered saline (PBS), and spectrophotometric grade chloroform (99.9% pure) used to prepare the phospholipid solution were from Sigma-Aldrich. Doxorubicin hydrochloride (DOX, purity 97.9%) was purchased from Supelco. The chemical structures of DOX and CR are shown in Scheme 1. An aqueous solution of PBS and PBS containing DOX (1 µM), CR (2 µM), or a DOX + CR mixture (1:1 *v*/*v* ratio) was used as subphases. PBS buffer was prepared using ultrapure water (MilliQ, Millipore) with a resistivity of 18 MΩ∙cm and a surface tension of 72.8 mN∙m^−1^ at 20 °C. 

### 4.2. Langmuir Trough Experiments

The measurements of surface pressure (*Π*)–area (*A*)isotherms were carried out with a KSV 2000 Langmuir balance (KSV Instruments, Helsinki, Finland). The isotherms were measured for the lipid monolayer formed on PBS, PBS/DOX, PBS/CR, and PBS/DOX + CR subphases. Surface pressure was monitored using a platinum Wilhelmy plate. A thermostated Teflon trough (58 × 15 × 1 cm) was equipped with two hydrophilic Delrin barriers. Before each measurement, the subphase surface was cleaned by sweeping and suction. The lipid solutions were spread with a Hamilton syringe on the subphase surface and left for 15 min to allow solvent evaporation and to reach an equilibrium state of the monolayer. All isotherms were recorded upon the symmetric compression of the monolayer at a constant rate of 5 mm min^−1^ barrier^−1^. Each compression isotherm was performed at least three times. The accuracy of the results was ±0.1 Å^2^ for area per lipid (APL) and ±0.01 mN∙m^−1^ for surface pressure. The *Π*–*A* isotherms were recorded at 37 ± 0.1 °C. The subphase temperature was maintained constant by a Lauda RE 104 thermostat. 

The surface pressure–area isotherms allowed determining the compressibility modulus (*C*_s_^−1^ = −*A*(d*Π*/d*A*)_T_) [57,58,59]. The collapse pressure *Π*_coll_ was determined directly from the compression isotherms [60,61]. 

### 4.3. Computational Details

Models of POPC bilayers with 100 and 225 lipids per leaflet, solvated with c.a. 30,000 and 66,000 water molecules, respectively, were prepared in three copies with Membrane Builder [62,63] functionality from CHARMM-GUI website [64] and equilibrated for 100 ns. Initial structures of CR, DOX, and DOX + CR clusters were cut from our previous simulations of DOX + CR aggregation in water [25]. DXP and DXP + CR clusters were prepared from DOX and DOX + CR clusters by protonating the NH_2_ group in DOX with the use of psfgen plugin in VMD [65]. All-atom CHARMM36 lipid force field [66] was used for POPC. Parameters for CR, DOX, and DXP were determined on the ground of CHARMM36 general force field for small molecules [67]. Missing parameters for DOX and CR were taken from our previous studies [25,35]. For DXP, atom types (Figure S14) as well as bonding and VDW parameters were assigned with the Ligand Reader & Modeler [68] functionality from CHARMM-GUI. ESP-derived partial charges (MK scheme [69]) calculated at B3LYP/6-31G* level of theory were used for this molecule. This approach was validated by checking the reproduction of the QM interaction energy profiles between DXP and a single water molecule. This test was performed in order to ensure the compatibility of the employed nonbonding parameters with the remaining components of the force field. The procedure for generating the interaction energy scans was adopted from the protocol proposed by Mayne at al. [70] for the parametrization of new molecules in CHARMM force fields. HF/6-31G* method was used for calculating the QM interaction energies. Gaussian09 suite was used for all quantum calculations [71]. MM energies were calculated with the NAMDEnergy tool available in VMD [65]. The results are presented in Figure S15. It can be seen that the reproduction of the QM interaction energy profiles is very good. Additional validation of the DXP parametrization was obtained by comparing the MM-optimized geometry with the MP2/6-31G* results. A good reproduction of the MP2 geometry was obtained (RMSD = 0.563, Figure S16). For water, the TIP3P model [72] was adopted in all simulations.

All molecular dynamics simulations reported in this study were conducted at *T* = 310 K and *p* = 1 atm. A semi-isotropic *N*p*T* ensemble with a constant ratio of *x* and *y* box dimensions was used. Temperature and pressure were controlled with Langevin thermostat and barostat, respectively. In all simulations, a timestep of 2 fs was used, and bonds with hydrogen atoms were constrained. A cutoff of 12 Å with switching from 10 Å was used. PME was used to include long-range electrostatic interactions [73]. All production MD simulations were performed with NAMD 2.14 package [74]. All user-defined forces, including harmonic wall potentials used to keep clusters whole and steered dynamics, were introduced through the collective variables module [75]. All visualization was performed in VMD [65].

In the adsorption simulations, either single molecules or small clusters (2–6 molecules) of CR, DOX, and DXP were placed symmetrically on both sides of pre-equilibrated POPC membranes with 100 lipids per leaflet (one on each side). For the largest clusters (CR3, DOX3, and DOX4CR2), additional models were built with a larger bilayer of 225 lipids per leaflet. The total charge of the systems containing CR or DXP was neutralized with NaCl. Three starting structures were built for each system and simulated independently. Initial configurations of selected replicas are shown in Figure S2. In order to keep the clusters in the vicinity of the bilayer, a harmonic wall potential was applied. Such a simulation setup was chosen on the basis of our previous experience with DOX + CR systems [25,35]. Due to only the low–moderate solubility of the studied compounds in water, low concentrations had to be used in the simulations. For example, neutral doxorubicin has a solubility of c.a. 0.3 mg·mL^−1^ [36], which corresponds to 1 DOX molecule for c.a. 70,000 water molecules. Due to the fact that doxorubicin and CR readily aggregate in water, the standard approach of inflating the concentration could not be used. Instead, single molecules and clusters were used. Wall potentials were then used to reduce unnecessary computational cost by eliminating configurations in which guest species are far in the aqueous phase and do not interact with the membrane. The potential was triggered when the position of the center of mass of the CR/DOX/DXP molecule–cluster along the *z* axis exceeded ±50 Å. Since the bilayer was centered at *z* = 0, this setup corresponded to constraining the CR/DOX/DXP distance from the bilayer center to 50 Å. The potential only affected the guest molecules and had no effect on either the lipids or water. For this purpose, the harmonic wall bias in the colvars module of NAMD was used. The bias was evaluated every 10 steps, and the force constant was equal to 10 kcal·mol^−1^·Å^−2^. In the case of DOX, an additional set of simulations were performed in which the harmonic wall potential was also applied to prevent cluster decomposition. In clusters with 2 DOX molecules, the distance between their COM was kept below 12 Å. In clusters with 3 molecules, the average of 3 distances between the individual pairs of molecules was kept below 15 Å. Such boundary distances were large enough to allow for temporary cluster loosening and reorganization but prevented them from falling apart. The bias was evaluated every timestep and the force constant was equal to 50 kcal·mol^−1^·Å^−2^. In this way, we were able to improve the sampling of interactions between the bilayer and DOX clusters, without the need to extend the simulations to very long time scales. Simulations were carried out for 280–520 ns. For the analyses of *R*_COM_(*z*) and interaction energies, whole adsorption trajectories, sampled every 1 ns, were used. Density profiles (Figure 2, Figures S7 and S8) were calculated with the first 50 ns of simulations discarded. Interaction energies were obtained by calculating the electrostatic and van der Waals contributions to the potential energy function along the obtained trajectories. Pair energies were summed for atom pairs belonging to the guest molecules and all lipids–water molecules. The force field parameters and cutoff scheme were the same as in the simulations. PME was used to include long-range electrostatic interactions. NAMDEnergy plugin from VMD was used to perform the calculation. Other quantities were calculated with VMD and self-developed scripts. 

Starting structures for the simulations of DOX/DXP/CR clusters inside the POPC membrane were generated by manually inserting guest species into the bilayer with moving harmonic restraints. Three systems were prepared for every cluster composition that exhibited bilayer entry in the adsorption simulations. The only omitted cluster was the CR3 system, because simulations with this cluster and 100 lipids/leaflet bilayer turned unstable. In addition, systems were prepared for DOX2, DOX3, DOX2CR1, and DXP2CR1 clusters. In the first two cases, the reason was to examine whether larger clusters will disintegrate in the membrane. In the last case, the reason was to check whether interactions between the membrane and the cluster change for a cluster with zero net charge. The DOX2CR1 system was added for comparison with DXP2CR1. The colvars module of NAMD was used for preparing the structures. Guest species were pulled from the center of the upper water slab to the center of the bilayer during the first 30 ns of 50 ns long simulations. Umbrella sampling technique was used for this purpose. Due to a faulty steered harmonic potential in the 2.14 version of NAMD, 3.0b3 version had to be employed at this stage. The distance along the *z* axis between the COM of the guest cluster and COM of N atoms from the bilayer was constrained. The force constant was equal to 100 kcal·mol^−1^·Å^−2^. During the final 20 ns of each simulation, the position of the guest was constrained in the bilayer center. Additional wall potential, with the same parameters as described above for DOX, was applied throughout the simulation to prevent cluster decomposition. In the next step, restraints on the guest COM were lifted and clusters were allowed to reach a stable position inside the bilayer during 100–200 ns simulations. Finally, wall potentials keeping the clusters whole were also removed and the systems were simulated for 250–400 ns. At this stage, 2.14 version of NAMD was restored in order to maintain consistency with the adsorption simulations. Simulations were carried out for DOX2, DOX3, DXP1, DXP2, DXP3, DOX2CR1, DOX2CR1, DXP1CR1, and DXP2CR1 cluster compositions. Three versions of each system were prepared and simulated independently. Each version originated from a different system replica from adsorption simulations. The last 100 ns of each simulation, sampled every 100 ps, were used for analysis of APL, density profiles, and order parameters. VMD and self-developed scripts/programs were used for the calculations. When averaging the above quantities, data from adsorption simulations were also included. In this case, only systems in which cluster insertion occurred were analyzed. Parts of the simulation preceding cluster insertion were discarded. The next 50 ns of each simulation was considered the equilibration time and was also discarded. If the remaining simulation time was shorter than 100 ns, it was all used for analysis. Otherwise, the last 100 ns were averaged. Order parameters were calculated with the formula: $S_{CD}=0.5*\langle 3{cos}^{2}\theta -1\rangle$, where *θ* is the angle between a C-H bond and bilayer normal, and the averaging is performed over ensemble and simulation time [76].

## Figures and Tables

**Table 1:** Summary outcome of all-atom MD simulations of DOX, CR, and DOX + CR clusters’ adsorption to solvated POPC bilayers of 100 and 225 lipids per leaflet at *T* = 310 K.

System	No. of Lipids per Leaflet	No. of Guest Molecules per System	Fraction of Penetrating Guest Molecules	
DOX1	100	2	50%	Disintegrated in water, penetrated the bilayer as single molecules
DOX2	100	4	50%	
DOX3	100	6	0%	
DOX3	225	6	17%	
DXP1	100	2	33%	Remained aggregated in water, penetrated the bilayer in one piece
DXP2	100	4	33%	
CR1	100	2	50%	Remained aggregated in water, penetrated the bilayer in one piece
CR2	100	4	17%	
CR3	100	6	17%	
CR3	225	6	17%	
DOX1CR1	100	4	17%	Remained aggregated in water, DOX1CR1 penetrated the bilayer in one piece, disintegrated in the membrane
DOX2CR2	100	8	0%	
DOX4CR2	100	12	0%	
DOX4CR2	225	12	0%	
DXP1CR1	100	4	33%	Remained aggregated in water, DXP1CR1 penetrated the bilayer in one piece
DXP2CR1	100	6	0%	

## Data Availability

Data are contained within the article and Supplementary Materials.

## Supplementary Materials

The following supporting information can be downloaded at: https://www.mdpi.com/article/10.3390/molecules29112567/s1, Figure S1: Structure of the molecules examined in the study; Figure S2: Initial structures of systems studied in the adsorption simulations; Figure S3: Final snapshots from selected adsorption simulations; Figure S4: Variations in areas per lipid and *z*-positions, *R*_COM_(*z*), of DOX and DXP molecules and POPC bilayers in the course of adsorption simulations; Figure S5: Variations in areas per lipid and *z*-positions, *R*_COM_(*z*), of POPC bilayers and CR molecules in the course of adsorption simulations; Figure S6: Variations in areas per lipid and *z*-positions, *R*_COM_(*z*), of mixed clusters and POPC bilayers in the course of adsorption simulations; Figure S7: Density profiles along bilayer normal, *ρ*(*z*), obtained from simulations of one-component clusters’ adsorption to POPC bilayer; Figure S8: Density profiles along bilayer normal, *ρ*(*z*), obtained from simulations of mixed clusters’ adsorption to POPC bilayer; Figure S9: Snapshot sequences illustrating entry of one-component clusters of DXP2, CR2, and CR3 to POPC bilayer; Figure S10: Variations in interaction energies between DOX1CR1 and DXP1CR1 clusters and POPC bilayer or water for a cluster located in the aqueous phase; Figure S11: Variations in the sum of VDW and electrostatic nonbonding interaction energies between cluster molecules: DOX-CR in DOX1CR1 and DXP-CR in DXP1CR1 during adsorption simulations; Figure S12: Final snapshots from individual simulations of DXP2CR1 clusters inside POPC bilayer and areas per lipid averaged over last 100 ns of each simulation; Figure S13: Order parameter for *sn*-1 lipid chains in POPC bilayers with one component and mixed DOX/DXP/CR clusters inside; Figure S14: CHARMM general force field atoms types adopted for the protonated doxorubicin molecule; Figure S15: Scans of interaction energies between DXP and a water molecule obtained at HF/6-31G* level of theory and molecular mechanics with nonbonding force field parameters determined in this work; Figure S16: Comparison between geometry of protonated doxorubicin optimized at MP2/6-31G* level of theory and MM with nonbonding parameters from CHARMM-GUI and derived in this work.
