# Supplementary material for: Insights into Transfer of Supramolecular Doxorubicin/Congo Red Aggregates through Phospholipid Membranes
Source: Molecules. 2024 May 30;29(11):2567. doi: 10.3390/molecules29112567 (PMC11173802; doi:10.3390/molecules29112567)
Supplement: Supplementary file 1 [file molecules-29-02567-s001.zip › molecules-3032817-supplementary.pdf]

Supplementary Materials for:

Insights into Transfer of Supramolecular Doxorubicin/Congo Red Aggregates through Phospholipid Membranes

Anna Stachowicz-Kuśnierz *, Paulina Rychlik, Jacek Korchowiec and Beata Korchowiec *

Faculty of Chemistry, Jagiellonian University, Gronostajowa 2, 30-387 Krakow, Poland

(a) Neutral doxorubicin, DOX

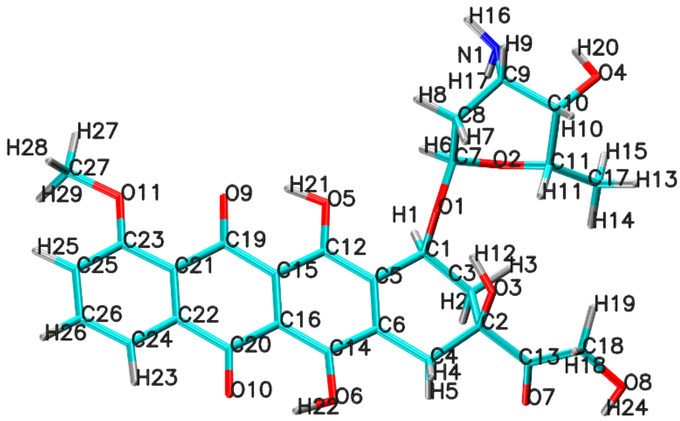

(b) Protonated doxorubicin, DXP

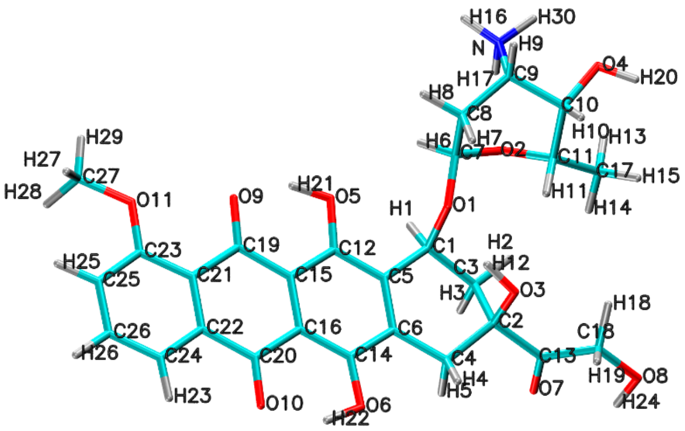

(c) Congo red, CR

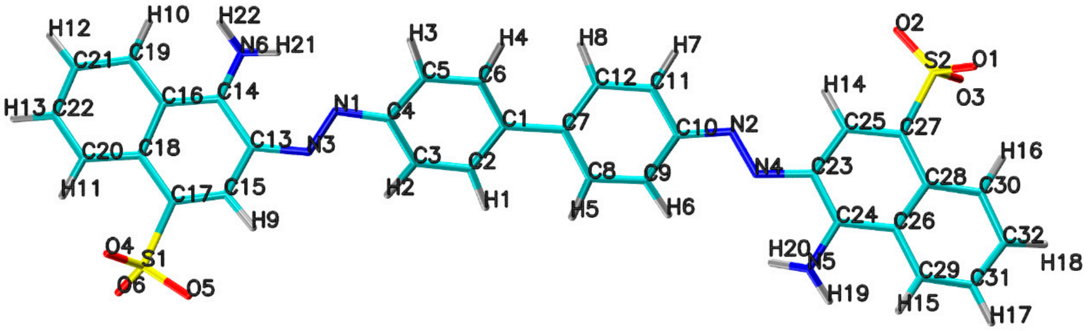

Figure S1. Structure of the molecules examined in the study: neutral doxorubicin (a), protonated doxorubicin (b), and Congo red (c). Color code: carbon – cyan, oxygen – red, nitrogen – blue, sulfur – yellow, hydrogen – grey. The adopted atom names are given as labels.

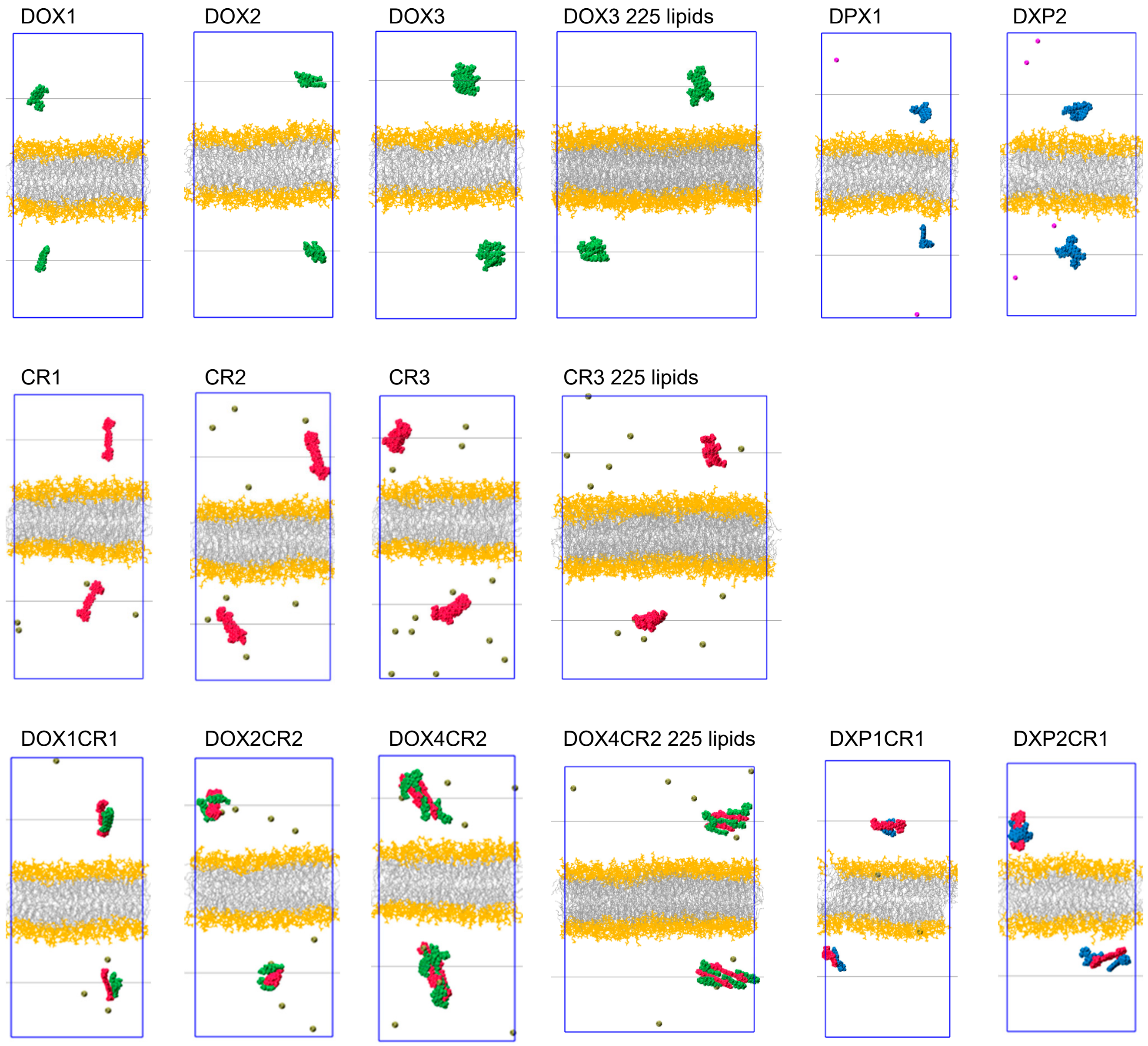

Figure S2. Initial structures of systems studied in the adsorption simulations. POPC bilayer of 100 or 225 lipids per leaflet is located in the center. Lipid headgroups are drawn with yellow lines, lipid chains are depicted in grey. Two identical clusters containing neutral doxorubicin (DOX, drawn with green spheres), protonated doxorubicin (DXP, blue spheres), or Congo red (CR, red spheres) were introduced to each system, one below and one above the membrane. Cluster composition, varying from one to six molecules per cluster, is given as numbers in each systems' label. Throughout the simulations, clusters were kept in vicinity of the bilayer with a harmonic-wall potential located at z = ±50 Å (grey horizontal lines). Counterions are shown with brown (Na^+) and magenta (Cl^-) spheres. Water is omitted for clarity.

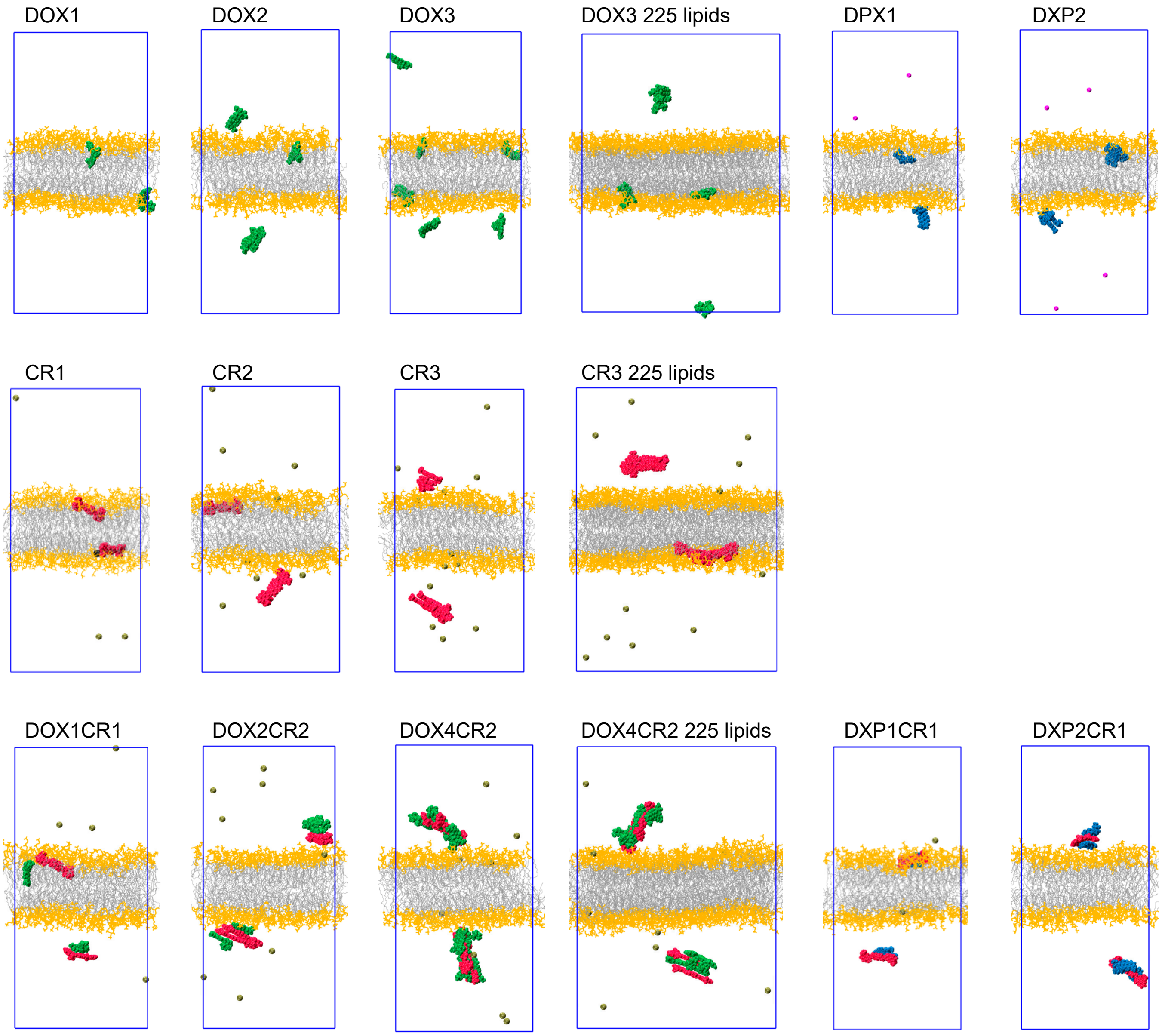

Figure S3. Final snapshots from selected adsorption simulations (280 – 520 ns, 310 K, 1 atm). Results for neutral doxorubicin (DOX, green spheres), protonated doxorubicin (DXP, blue spheres), Congo red (CR, red spheres), or mixed DOX/CR and DXP/CR clusters and POPC bilayers with 100 and 225 lipids per leaflet are shown. Cluster composition, varying from one to six molecules per cluster, is given as numbers in each systems' label. Lipid headgroups are drawn with yellow lines, lipid chains are depicted in grey. Na^+ and Cl^- counterions are depicted with brown and magenta spheres, respectively. Water is omitted for clarity.

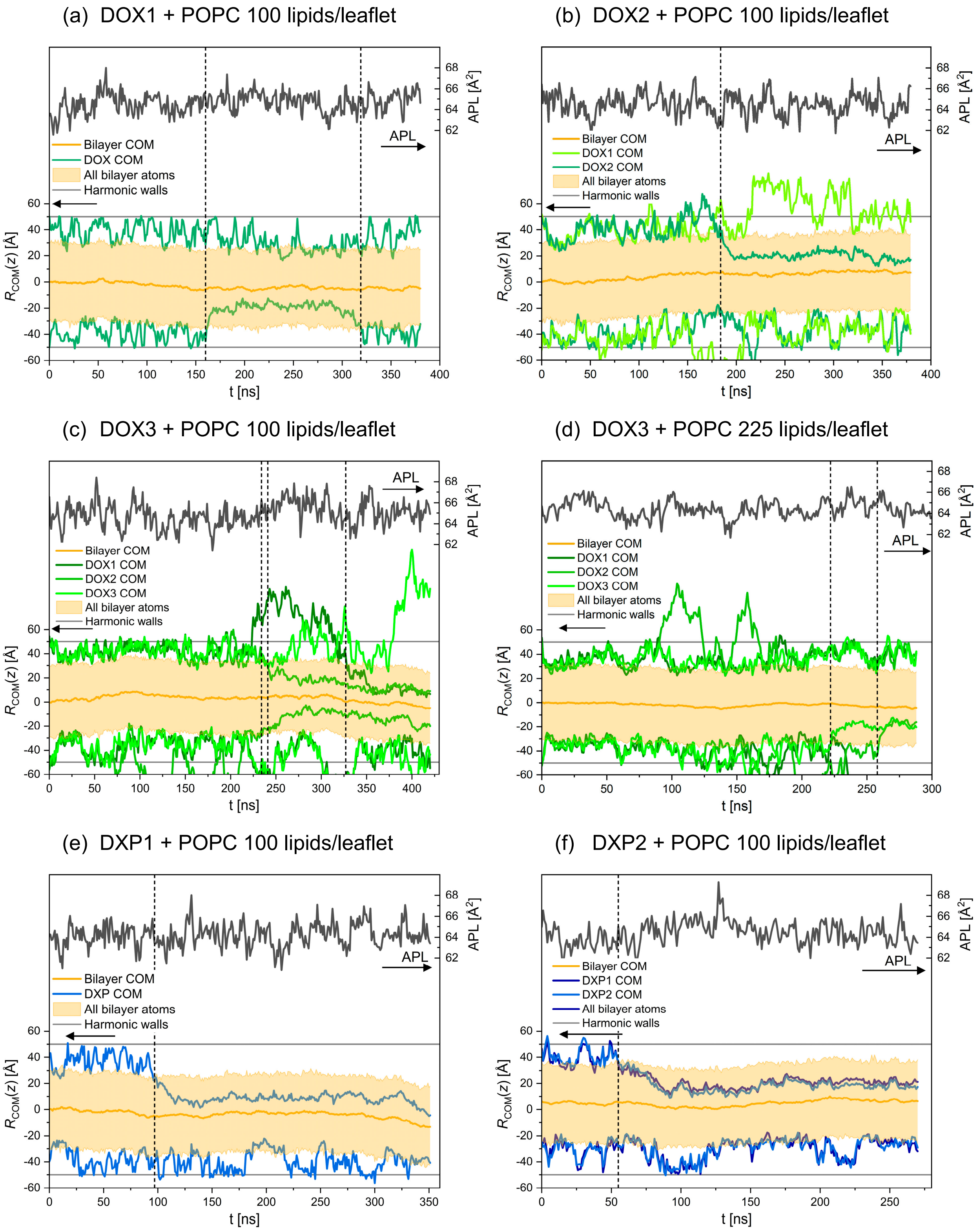

Figure S4. Variations in areas per lipid (APL, dark grey) and *z*-positions, $R_{COM}(z)$, of DOX (green) and DXP (blue) molecules and POPC bilayers (orange) in the course of adsorption simulations. Results are given for single molecules (a,e) and clusters of two (b,f) or three (c, d) guest molecules with bilayers of 100 (a-c, e, f) and 225 (d) lipids/leaflet. APL is plotted along axes on the right, and $R_{COM}(z)$ along axes on the left, as indicated by arrows. Location of harmonic-walls used to keep guest molecules in vicinity of the bilayers is shown with horizontal grey lines. Vertical dashed lines indicate the time of guest molecules entry into the bilayers.

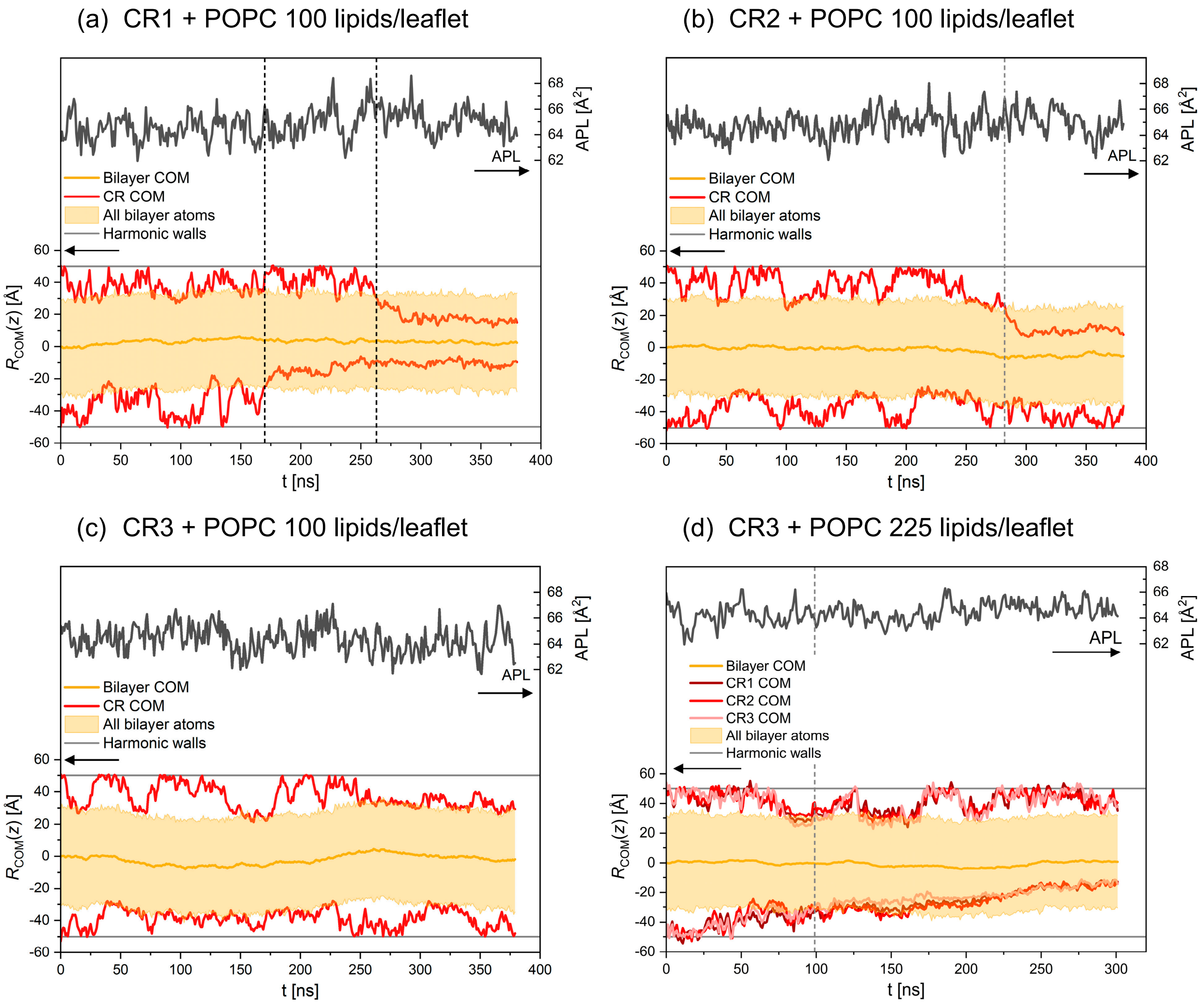

Figure S5. Variations in areas per lipid (APL, dark grey) and *z*-positions, $R_{COM}(z)$, of POPC bilayers (orange) and CR molecules (red) in the course of adsorption simulations. Results are given for single molecules (a) and clusters of two (b) or three (c, d) CR molecules with bilayers of 100 (a-c) and 225 (d) lipids/leaflet. APL is plotted along axes on the right, and $R_{COM}(z)$ along axes on the left, as indicated by arrows. Location of harmonic-walls used to keep guest molecules in vicinity of the bilayers is shown with horizontal grey lines. Vertical dashed lines indicate the time of guest molecules entry into the bilayers.

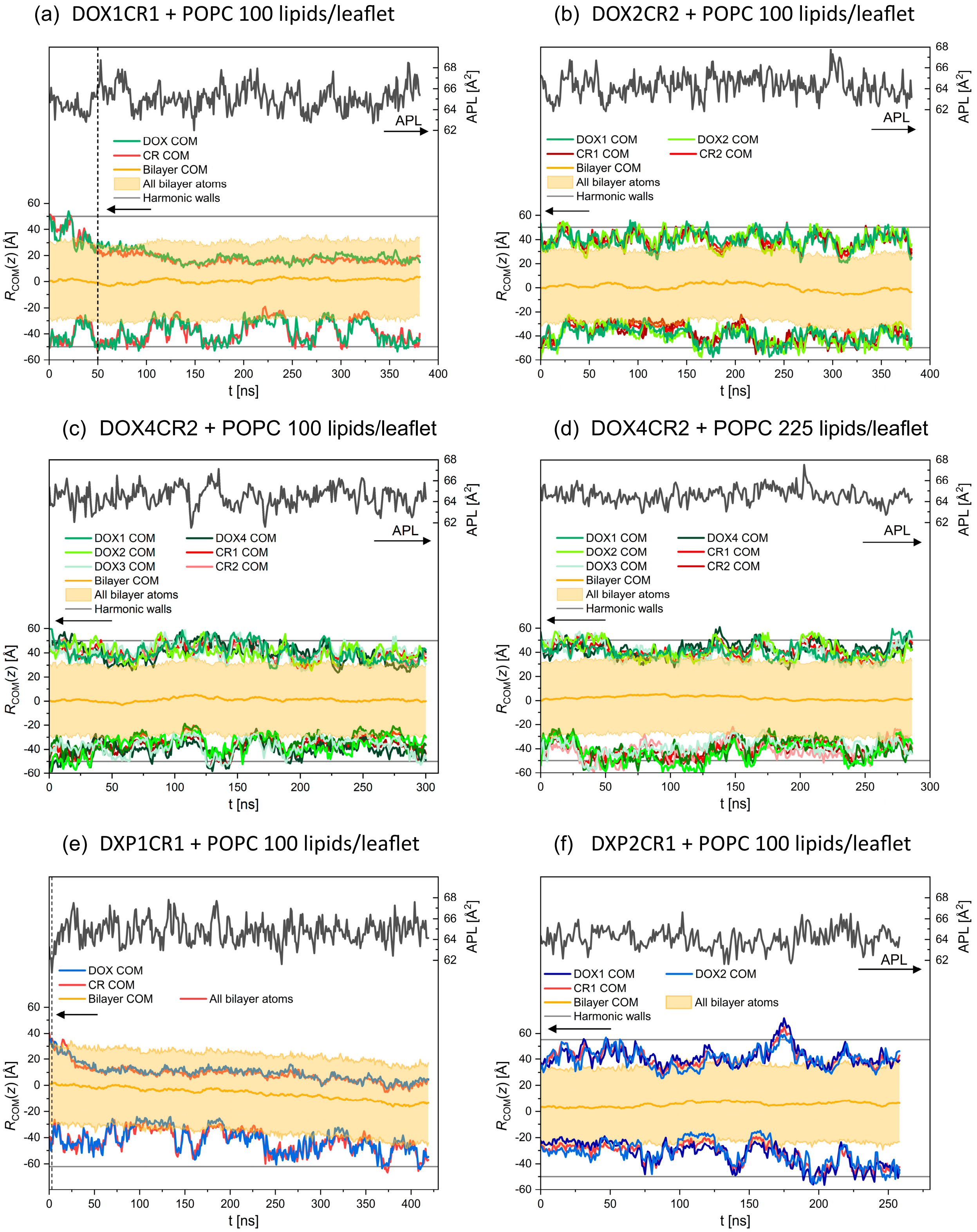

Figure S6. Variations in areas per lipid (APL, dark grey) and *z*-positions, $R_{COM}(z)$, of mixed clusters (DOX – green, DXP – blue, CR – red) and POPC bilayers (orange) in the course of adsorption simulations. Results are given for: mixed DOX/CR clusters composed of two (a), four (b), and six (c, d) molecules, and DXP/CR cluster composed of two (e) or three (f) molecules. APL is plotted along axes on the right, and $R_{COM}(z)$ along axes on the left, as indicated by arrows. Location of harmonic-walls used to keep guest molecules in vicinity of the bilayers is shown with horizontal grey lines. Vertical dashed lines indicate the time of guest molecules entry into the bilayers.

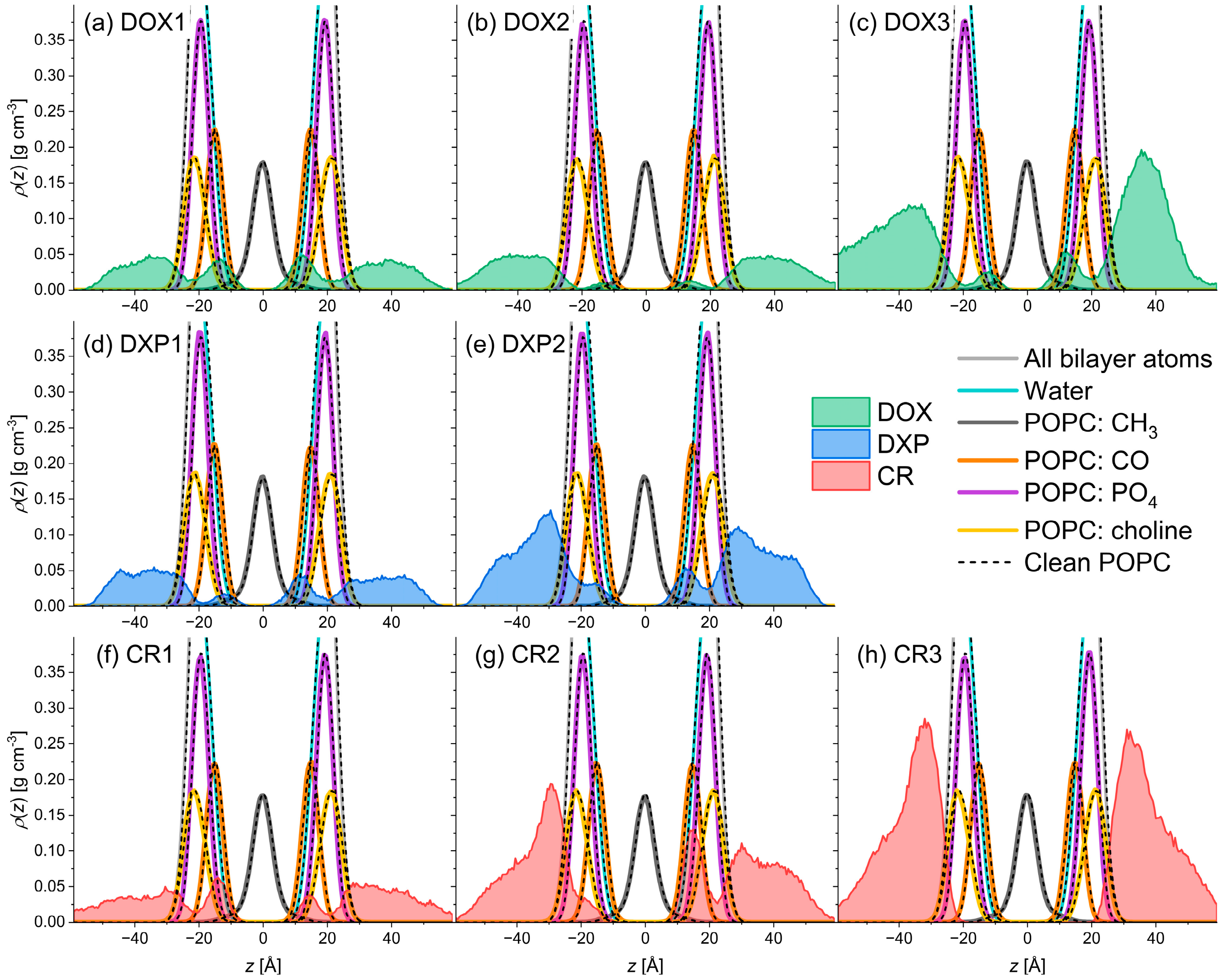

Figure S7. Density profiles along bilayer normal, $\rho(z)$, obtained from simulations of one-component clusters' adsorption to POPC bilayer (100 lipids/leaflet, 310 K, 1 atm). The plots were averaged for simulation times t > 50 ns. All curves are averages over three independent simulations. Colored solid lines represent water (cyan), all bilayer atoms (light grey), and selected bilayer components: *sn*-1 and *sn*-2 chain terminal CH_3 groups (dark grey), *sn*-1 and *sn*-2 carbonyl groups (orange), phosphate groups (magenta), and choline (yellow). Dashed black lines mark the profiles obtained for clean POPC systems. Shaded areas represent the profiles obtained for guest molecules: neutral doxorubicin (DOX, green), protonated doxorubicin (DXP, blue), and Congo red (CR, red). Cluster composition, varying from one to three molecules per cluster, is given as numbers in each systems' label. The profiles for guest molecules were magnified ten times.

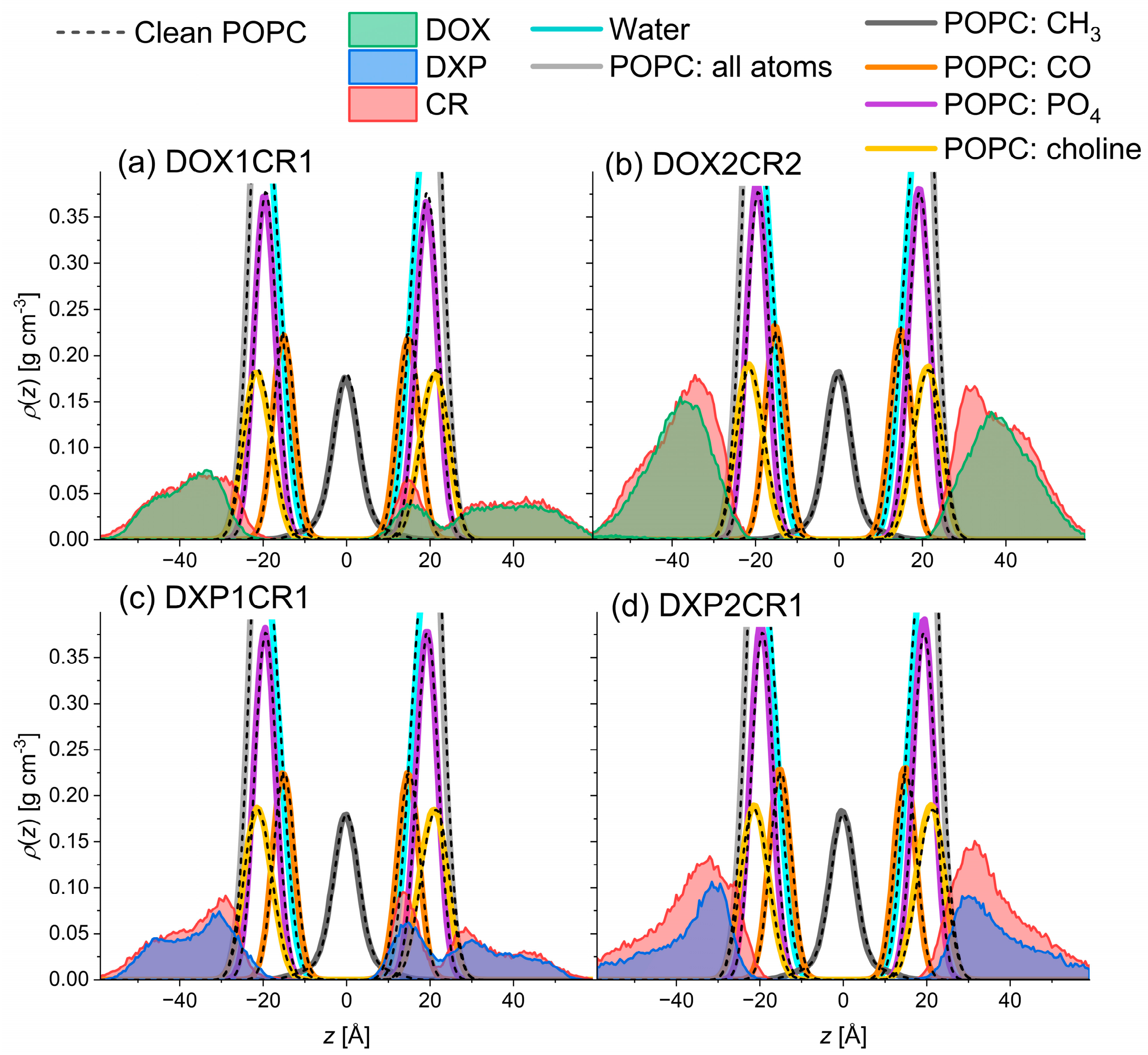

Figure S8. Density profiles along bilayer normal, $\rho(z)$, obtained from simulations of mixed clusters' adsorption to POPC bilayer (100 lipids/leaflet, 310 K, 1 atm). The plots were averaged for simulation times t > 50 ns. All curves are averages over three independent simulations. Colored solid lines represent water (cyan), all bilayer atoms (light grey), and selected bilayer components: *sn*-1 and *sn*-2 chain terminal CH_3 groups (dark grey), *sn*-1 and *sn*-2 carbonyl groups (orange), phosphate groups (magenta), and choline (yellow). Dashed black lines mark the profiles obtained for clean POPC systems. Shaded areas represent the profiles obtained for guest molecules: neutral doxorubicin (DOX, green), protonated doxorubicin (DXP, blue), and Congo red (CR, red). Cluster composition, varying from two to four molecules per cluster, is given as numbers in each systems' label. The profiles for guest molecules were magnified ten times.

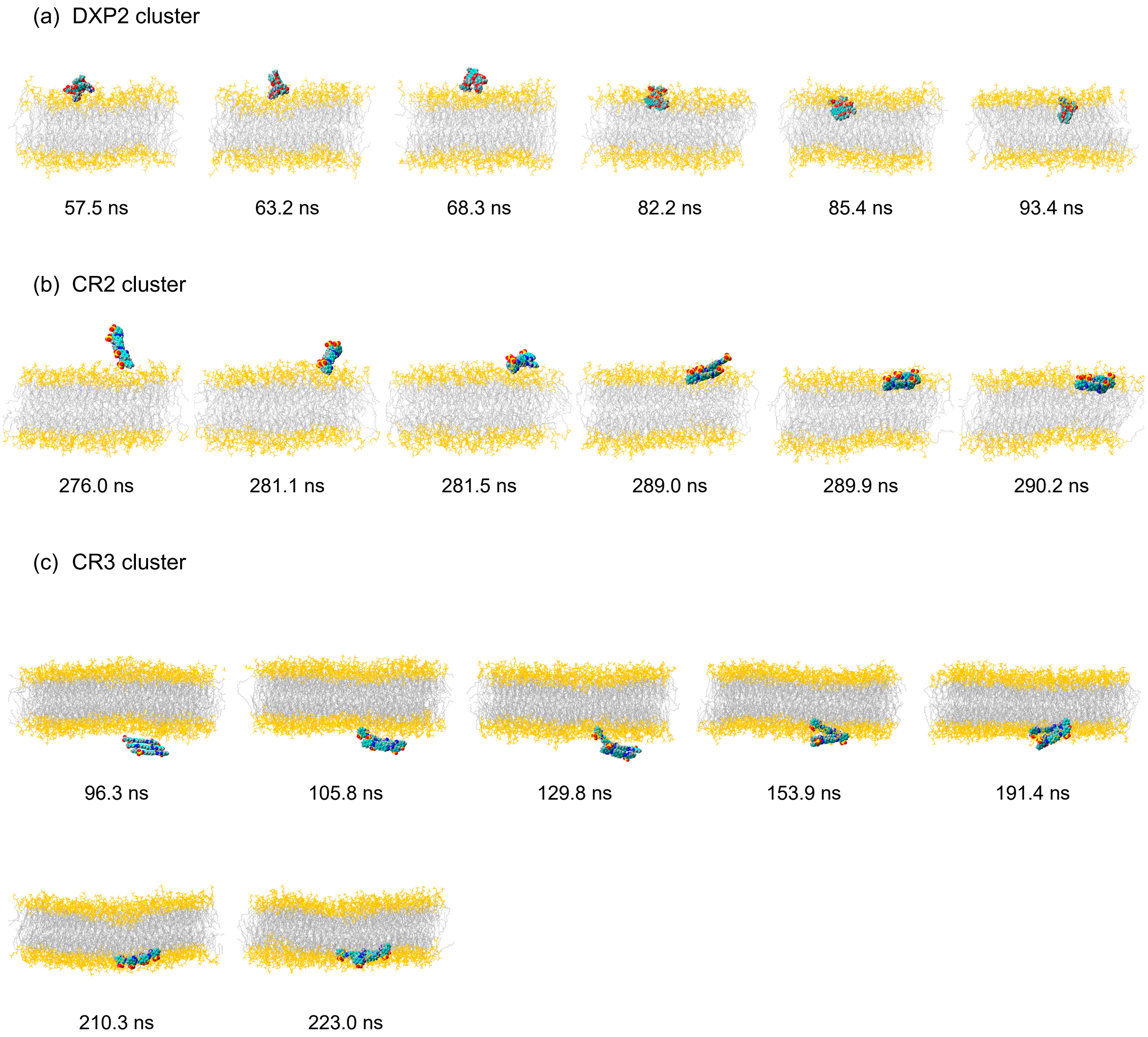

Figure S9. Snapshot sequences illustrating entry of one-component clusters of DXP2 (a), CR2 (b), and CR3 (c) to POPC bilayer with 100 (a,b) or 225 lipids/leaflet (c). Snapshots are labelled with a timestamp corresponding to the time elapsed from the beginning of each simulation. Lipid headgroups are drawn with yellow lines, lipid chains are depicted in grey. DXP, and CR are depicted with van der Waals spheres, colored according to element: carbon – cyan, oxygen – red, nitrogen – blue, sulfur – yellow, hydrogen – grey. Water, counterions, and the second guest cluster are omitted for clarity.

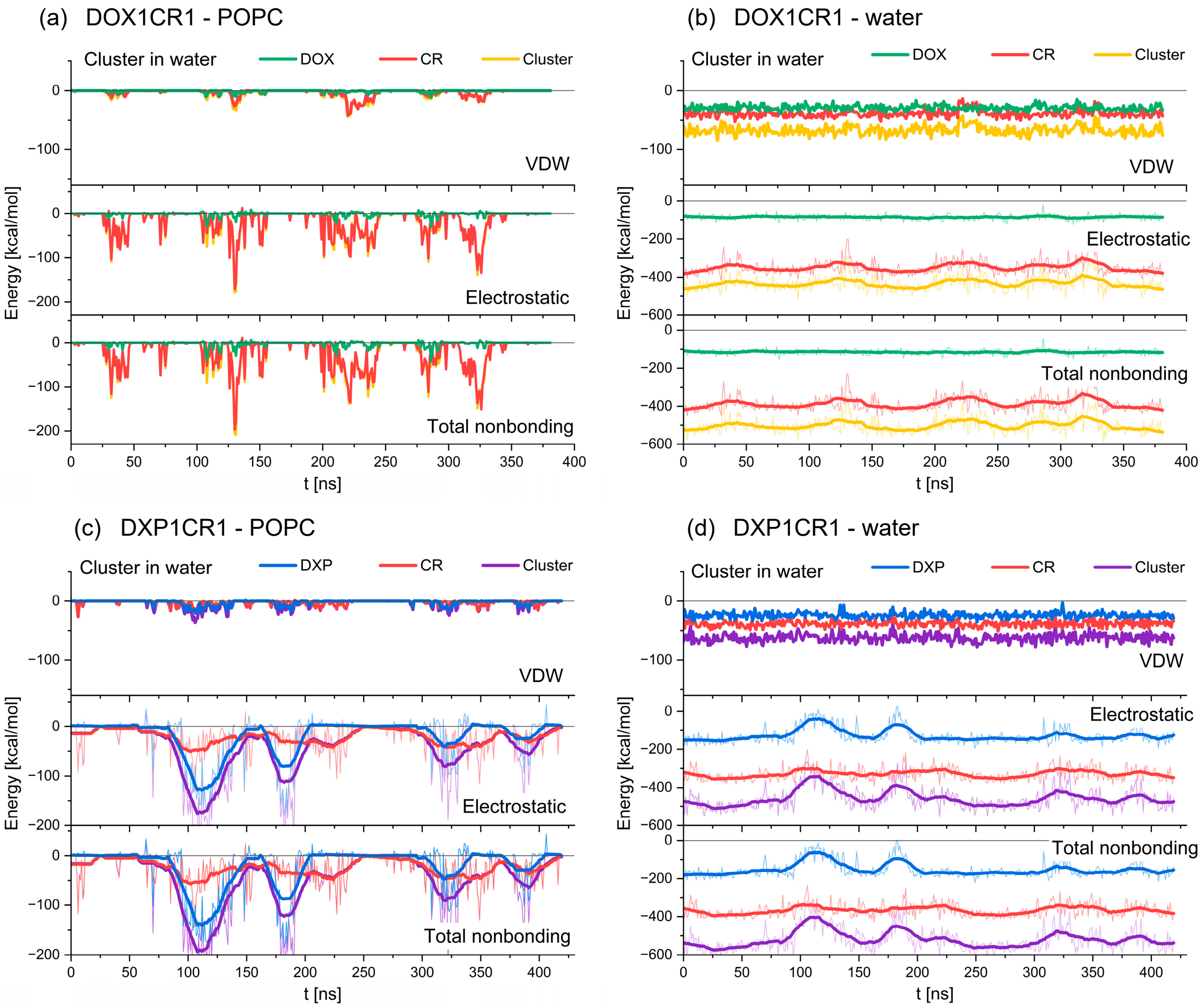

Figure S10. Variations in interaction energies between DOX1CR1 (a,b) and DXP1CR1 (c,d) clusters and POPC bilayer (a,c) or water (b,d) for a cluster located in the aqueous phase. Van der Waals (top) and electrostatic (middle) contributions, and their sum (bottom) are plotted separately. Each energy term is further split into individual DOX (green), DXP (blue), and CR (red) contributions, and their sum (yellow and violet). For electrostatic and total energies smoothed curves are plotted, due to large fluctuations. Unsmoothed energies are shown as lighter lines in the background.

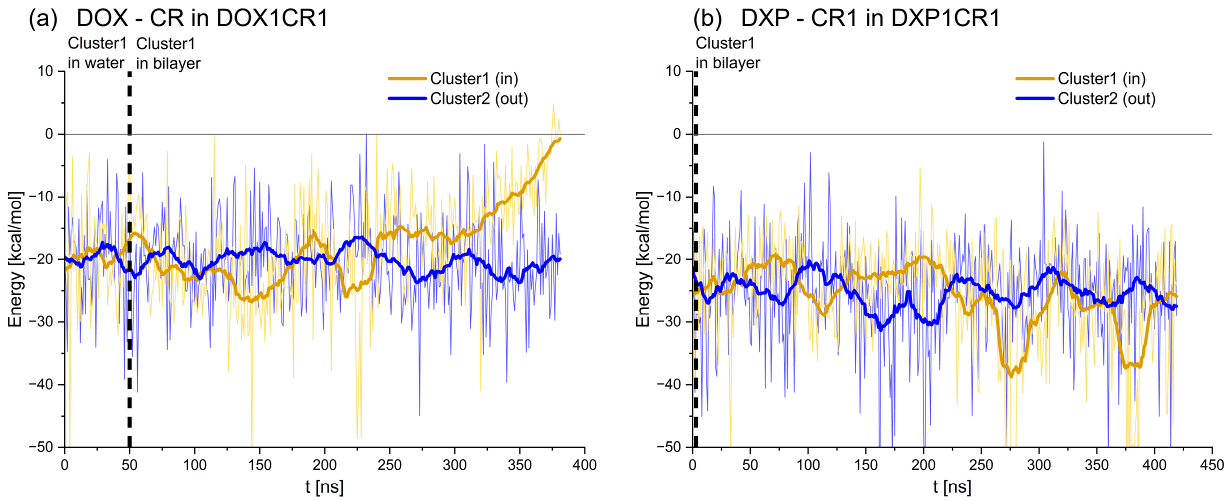

Figure S11. Variations in the sum of VDW and electrostatic nonbonding interaction energies between cluster molecules: DOX-CR in DOX1CR1 (a) and DXP-CR in DXP1CR1 (b) during adsorption simulations. Curves for clusters penetrating the bilayer (yellow) and remaining in water (blue) are compared. Due to large fluctuations, smoothed curves are plotted highlighted and unsmoothed energies are shown as lighter lines in the background. Vertical dashed lines mark the time when COM of the penetrating cluster crosses the membrane boundary.

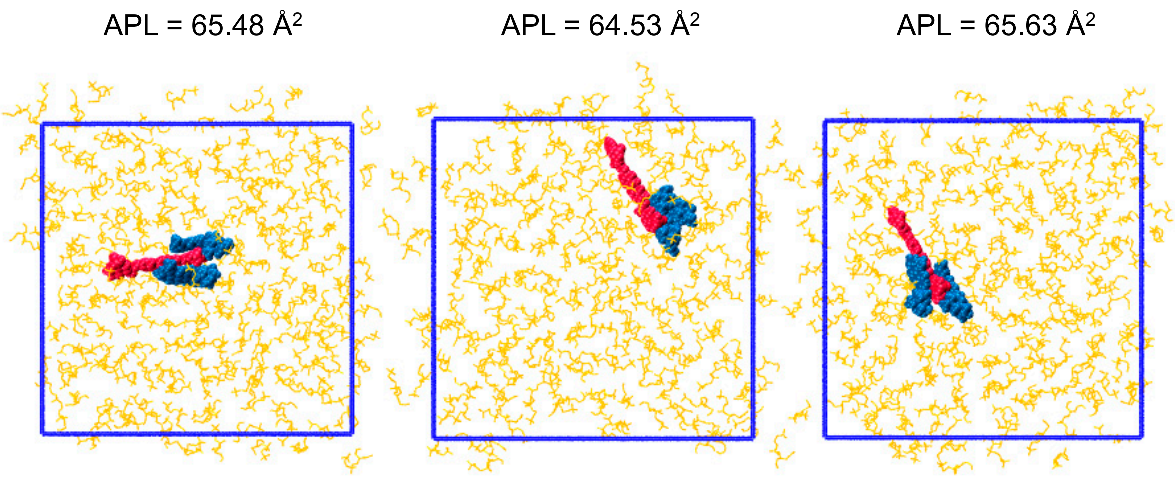

Figure S12. Final snapshots from individual simulations of DXP2CR1 clusters inside POPC bilayer and areas per lipid (APL) averaged over last 100 ns of each simulation. Lipid headgroups – yellow, Congo red – red, protonated doxorubicin – blue.

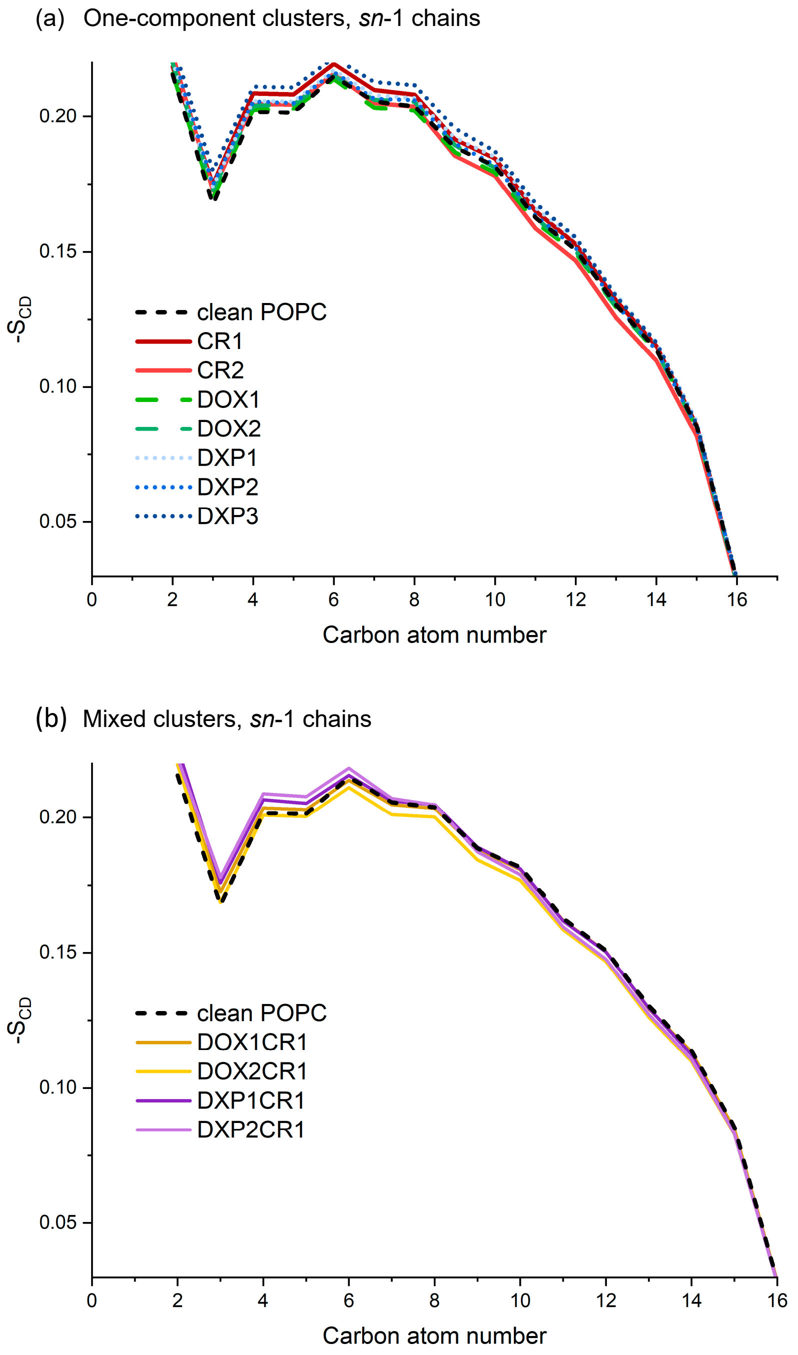

Figure S13. Order parameter for *sn*-1 lipid chains in POPC bilayers with one component (a) and mixed (b) DOX/DXP/CR clusters inside. The values were obtained by averaging over all systems with a given cluster inside the bilayer (3-6 systems). Results for clean POPC bilayer are shown with dashed lines.

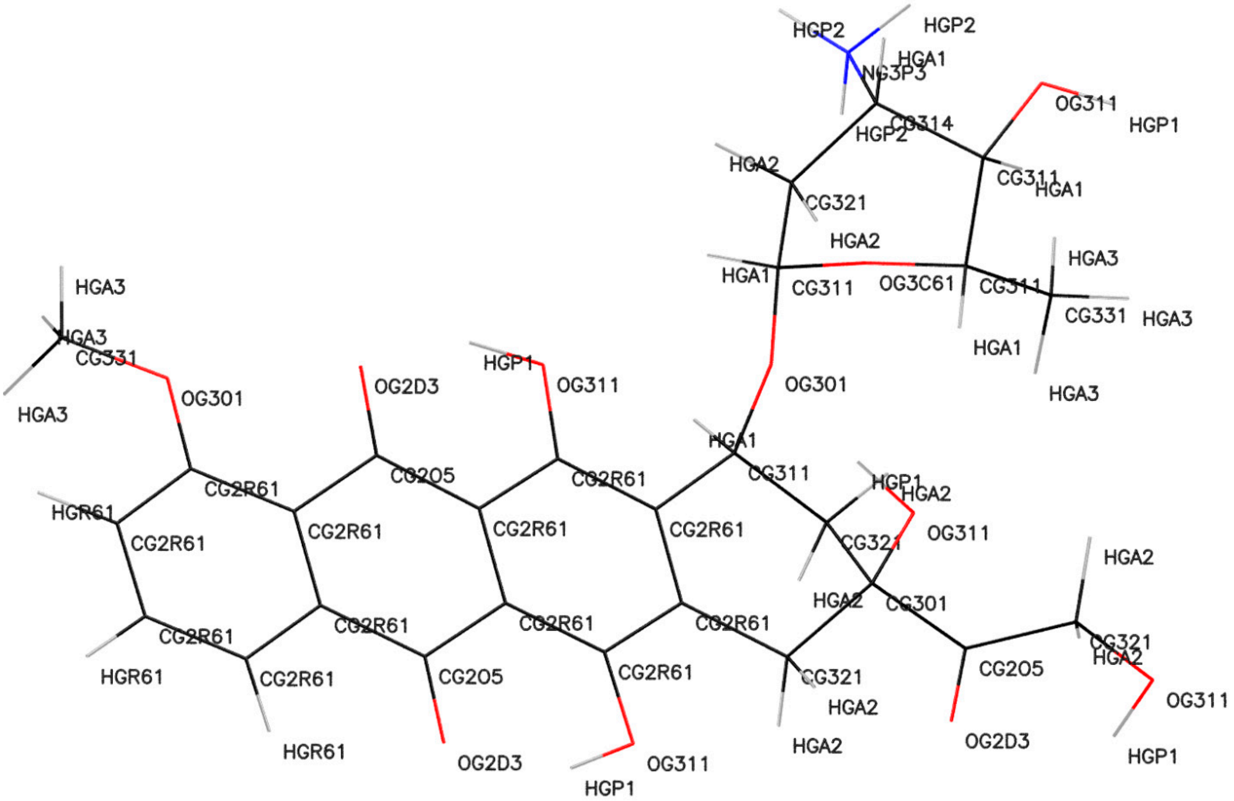

Fig S.14. CHARMM general force field atoms types adopted for the protonated doxorubicin molecule (DXP).

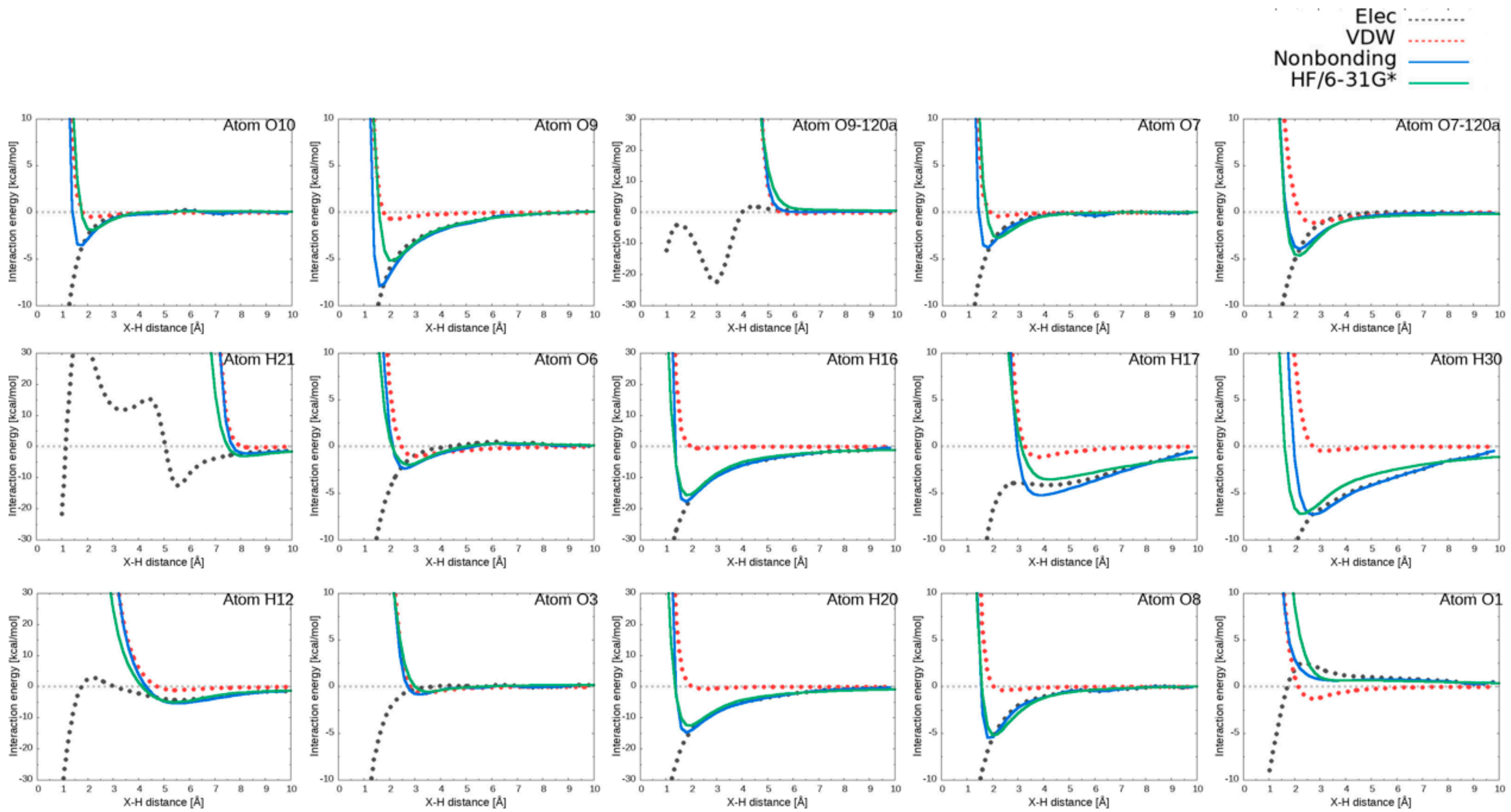

Figure S15. Scans of interaction energies between DXP and a water molecule obtained at HF/6-31G* level of theory (green) and molecular mechanics (blue) with nonbonding force field parameters determined in this work. MM energy was further divided into electrostatic (black) and van der Waals (red) contributions. The scans were obtained by placing a rigid water molecule (TIP3P geometry) in proximity of HB donor (Y) and acceptor (X) groups in an idealized hydrogen bonding geometry (X···H distance equal to 2.0 Å, X···H-Y angle equal to 180°). X···H distance and the orientation of the water molecule were then optimized at the HF/6-31G* level of theory. Finally, X···H distance was varied in the range 1-10 Å with a step of 0.2 Å to calculate the energy scan.

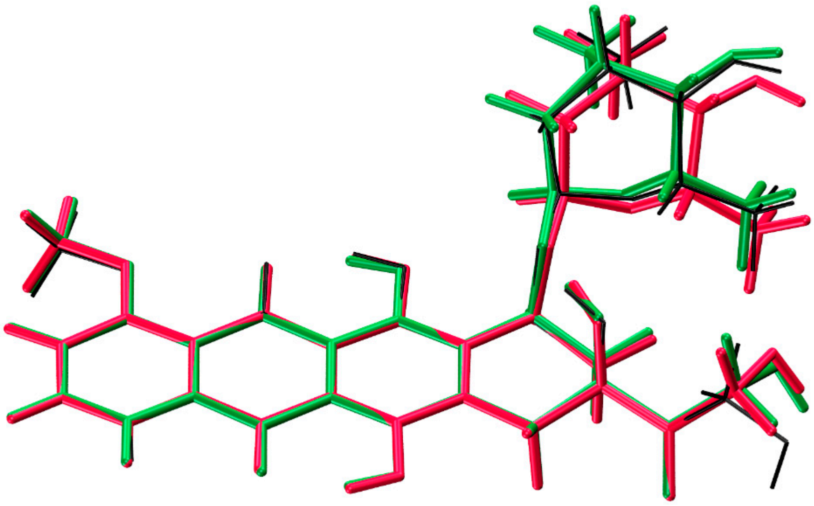

Figure S16. Comparison between geometry of protonated doxorubicin (DXP) optimized at MP2/6-31G* level of theory (black) and MM with nonbonding parameters from CHARMM-GUI (red) and derived in this work (green).
